# Switchbody, an Antigen‐Responsive Enzyme Switch Based on Antibody and Its Working Principle

**DOI:** 10.1002/advs.202508422

**Published:** 2025-09-15

**Authors:** Takanobu Yasuda, Yoshiyuki Ueno, Masahiko Taguchi, Naoya Tochio, Hiromasa Yagi, Shuma Yazaki, Ryoichi Arai, Bo Zhu, Takanori Kigawa, Hiroshi Ueda, Tetsuya Kitaguchi

**Affiliations:** ^1^ Laboratory for Chemistry and Life Science Institute of Integrated Research Institute of Science Tokyo Yokohama 226‐8501 Japan; ^2^ Graduate School of Life Science and Technology Institute of Science Tokyo Yokohama 226‐8501 Japan; ^3^ Institute of Multidisciplinary Research for Advanced Materials Tohoku University Sendai 980‐8577 Japan; ^4^ Laboratory for Cellular Structural Biology Center for Biosystems Dynamics Research RIKEN Yokohama 230‐0045 Japan; ^5^ Department of Applied Biology Faculty of Textile Science and Technology Shinshu University Ueda 386‐8567 Japan; ^6^ Division of Life Innovation Institute for Biomedical Sciences Interdisciplinary Cluster for Cutting Edge Research Shinshu University Matsumoto 390‐8621 Japan

**Keywords:** antibodies, enzyme switch, immunosensors, molecular dynamics, protein structures

## Abstract

An enzyme switch, termed “Switchbody”, is developed by fusing an antibody with a fragment of a split enzyme for the precise regulation of enzyme activity in response to an antigen. A luciferase‐based Switchbody is engineered by fusing the NanoLuc luciferase fragment HiBiT to the N‐terminus of an antibody. The enzyme activity of the Switchbody increases upon the addition of an antigen in a dose‐dependent manner in the presence of the complementary fragment LgBiT and its substrate furimazine, demonstrating the potential of the luciferase‐based Switchbody as a biosensor. As its working principle, ELISA shows that the interaction between HiBiT and LgBiT is facilitated by antigen binding. Moreover, X‐ray crystallography and NMR reveal the heterogeneous trapped state of the HiBiT region and an increasing motility of HiBiT region upon antigen binding, respectively. MD simulations and luminescence measurements show that antigen disrupted the trapping of HiBiT in the antibody, enabling its release. By applying this “Trap and Release” principle to Protein M, an antibody‐binding protein, label‐free IgG antibodies are successfully converted into bioluminescent Switchbodies. This adaptable Switchbody platform has the potential to expand switching technology beyond luciferase to various other enzymes in the future.

## Graphical abstract

This paper presents an antibody‐based enzyme switch, termed a “Switchbody”, which is fused with an enzyme‐derived fragment to the N‐terminus of an antibody. As its working principle, the fused fragment is trapped within the antibody and released upon antigen binding, leading to enzyme reconstitution and a subsequent increase in enzyme activity. This “Trap and Release” principle provides a framework for designing next‐generation enzyme switches.

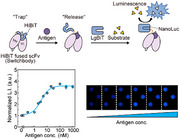

## 1 Introduction

Proteins undergo dynamic structural changes in response to various external stimuli, such as ligand binding, shifts in membrane potential, fluctuations in pH or temperature, and post‐translational modifications. These structural changes orchestrate various cellular physiological functions, including metabolism, signal transduction, and cytoskeletal reorganization. In other words, proteins work as molecular switches that dynamically respond to external stimuli. Therefore, by artificially manipulating the on/off states of a protein like a switch, these cellular functions can be controlled as desired or visualized at a high spatiotemporal resolution.^[^ ^1^, ^2^ ^]^

Several protein‐based switches have been engineered to manipulate their activity by external stimuli. Examples include the light‐sensitive LOV2 domain of the *Avena sativa* phototropin 1 (AsLOV2) fusion protein to control effector access to proteins of interest,^[^ ^3^, ^4^, ^5^ ^]^ designer receptors exclusively activated by designer drugs (DREADD) to activate G protein‐coupled receptors (GPCRs) with synthetic ligands,^[^ ^6^, ^7^ ^]^ the Tet‐On/Off system to precisely regulate gene expression,^[^ ^8^ ^]^ rapamycin‐induced FKBP‐FRB dimerization to induce cooperative and hierarchical protein functions,^[^ ^9^, ^10^ ^]^ and fluorescent protein‐based biosensors using protein‐protein interactions^[^ ^11^, ^12^ ^]^ or conformational changes^[^ ^13^, ^14^ ^]^ to visualize the dynamics of target molecules. These switches are widely used and have been successful in applications such as controlling cellular physiological functions, molecular imaging, and diagnostics. However, they are limited by their inability to accommodate desired stimuli, since the switch region of the protein usually depends on the stimuli it originally receives.

To address this limitation, protein switches employing antibodies have been developed. The ability of antibodies to recognize targeted molecules and carry the high affinity and specificity for antigens make them ideal as switch components. Several advanced antibody‐based switches have been reported, including chimeric antigen receptors (CARs) for cancer treatment,^[^ ^15^ ^]^ target‐dependent RNA polymerase (TdRNAP) for transcriptional regulation,^[^ ^16^ ^]^ and antibody‐based homogeneous biosensors for applications such as in situ immunoassays,^[^ ^17^, ^18^, ^19^, ^20^ ^]^ monitoring protein production,^[^ ^21^ ^]^ and live‐cell imaging.^[^ ^22^, ^23^, ^24^ ^]^

Split enzymes frequently serve as the functional domains driving the output of these antibody‐based switches. To date, these antigen‐triggered enzyme switches have been engineered via integration of various split enzymes such as RNA polymerase,^[^ ^16^ ^]^ β‐galactosidase,^[^ ^25^ ^]^ β‐lactamase,^[^ ^26^, ^27^ ^]^ and luciferase^[^ ^20^, ^28^, ^29^ ^]^ with antibodies, allowing them to control transcription and to quantify antigen concentration through colorimetric or bioluminescence reactions using reconstitution of split enzymes induced by antigen‐antibody binding. However, the reconstitution of split enzymes typically requires either the use of two antibodies or the antigen‐induced association of V_H_ and V_L_ domains. Since successful reconstitution requires precise control over protein concentrations, binding orientation, and the spatial distance between the two protein components, we hypothesized that split enzyme reconstitution triggered by antigen binding to a single antibody is a viable strategy to overcome these challenges.

**Figure 1:**
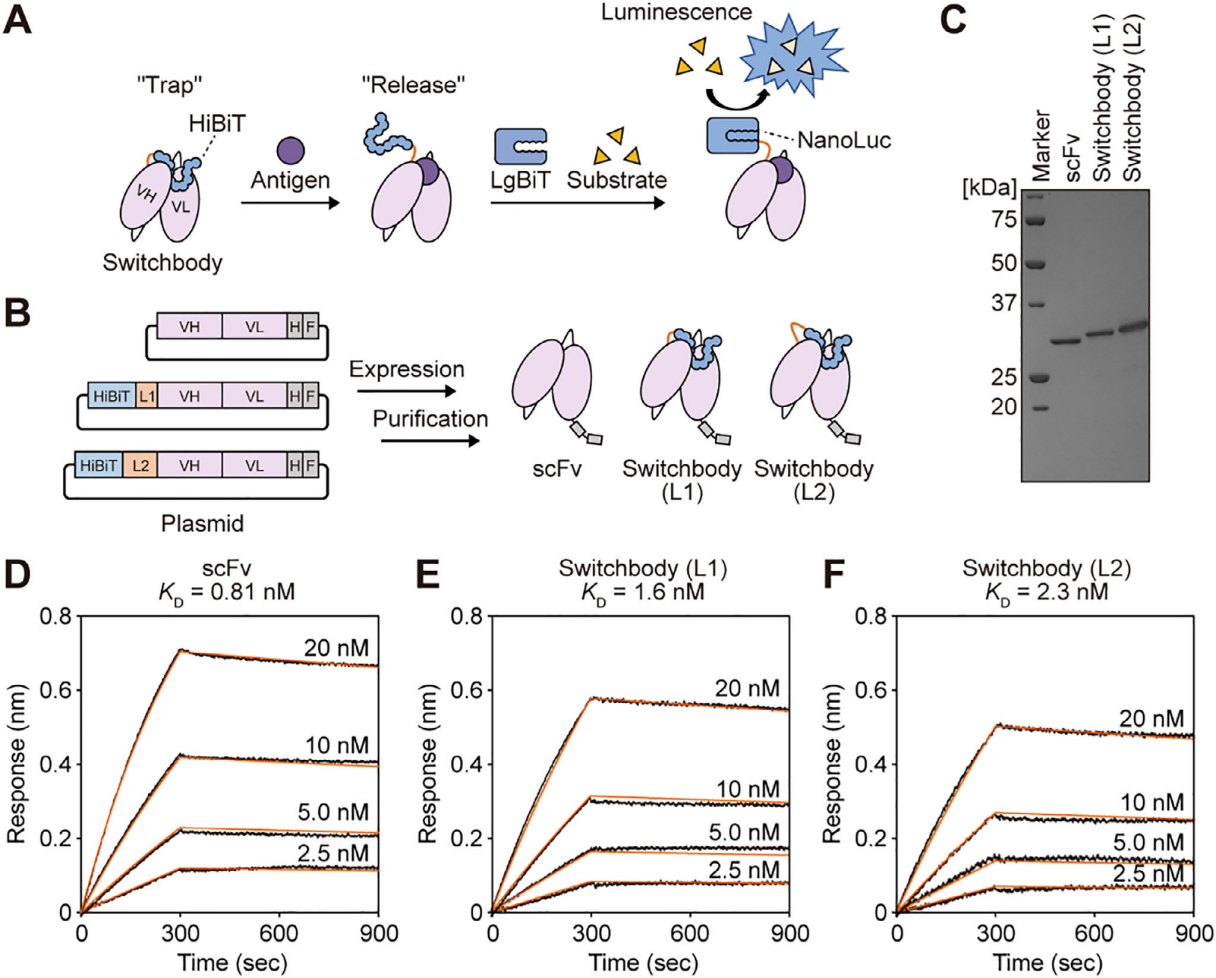
Design and fabrication of Switchbody. A) Schematic illustrating the concept of antigen detection by Switchbody. B) Schematic illustration of the fabrication process for scFv and Switchbodies. L1: (G_3_S) linker, L2: (G_3_S)_2_ linker, H: 6×His‐tag, F: FLAG‐tag. C) SDS‐PAGE analysis of purified scFv and Switchbodies. D–F) Binding activity of scFv and Switchbodies to immobilized biotinylated BGP‐C11 antigen, examined by BLI. Black and orange lines indicate row data and global fitted curves, respectively.

We previously attempted to develop a luciferase‐based reporter using a single‐chain variable fragment (scFv) conjugated with HiBiT, an 11‐amino‐acid fragment derived from a bioluminescent enzyme NanoLuc,^[^ ^30^ ^]^ which interacts with high affinity with the large complimentary fragment, LgBiT, of NanoLuc. During these attempts, we serendipitously discovered that the addition of an antigen in the presence of LgBiT and its substrate increased the bioluminescence of the HiBiT‐fused scFv. We exploited the concept of this discovery and developed an enzyme switch, the bioluminescent “Switchbody”. The HiBiT region is trapped in the antibody fragment and released upon antigen binding (**Figure** **1**A), a process we investigated via ELISA, X‐ray crystallography, NMR, and molecular dynamics simulations. Furthermore, we developed an antibody‐binding protein M (PM)^[^ ^31^ ^]^‐based probe carrying HiBiT and used it to convert commercially available antibodies, such as anti‐TARGET‐tag IgG and anti‐Thyroxine IgG into enzyme switches, implying that a myriad of molecules can potentially be used as inputs for bioluminescent enzyme switches. The innovative principle of this enzyme switch, in which a luciferase‐derived fragment is trapped in an antibody and released upon antigen binding, holds the potential to revolutionize immunoassays with multiplex detection through distinct enzymes and substrates, to control various cellular physiological functions, and to facilitate next‐generation drug delivery systems with precise regulation of the timing and location of therapeutic release.

## 2 Results and Discussion

### 2.1 Preparation of HiBiT‐Fused anti‐BGP scFvs, Switchbodies

To investigate whether fusion of the HiBiT in the vicinity of the antigen‐binding site of an antibody result in antigen‐dependent bioluminescence increase, we prepared scFvs fused with HiBiT at the N‐terminus via either a G_3_S or (G_3_S)_2_ linker (Figure 1B). As a reason for the choice of these linkers, we considered that flexible linkers more suitable than rigid linkers for antigen‐dependent HiBiT release and its transition to a higher motile state, leading to reconstitution of NanoLuc. We employed the scFv of the anti‐human osteocalcin (Bone Gla Protein, BGP) antibody KTM219^[^ ^32^ ^]^ as a model antibody due to our familiarity with its properties and crystal structure.^[^ ^17^ ^]^ When the scFv and these HiBiT‐fused scFvs (dubbed “Switchbodies”) were expressed in the *Escherichia coli* and purified, we observed the expected bands of scFv, Switchbody (L1) carrying G_3_S linker, and Switchbody (L2) carrying (G_3_S)_2_ linker by SDS‐PAGE analysis (Figure 1C). We then performed a Bio‐Layer Interferometry (BLI) to confirm the affinity of recombinant scFv and Switchbodies. Switchbodies showed slightly lower affinity compared to the scFv (Figure 1D–F; Table S1, Supporting Information), suggesting that HiBiT was located at the vicinity of the antigen‐binding site and partially obstructed antigen binding via steric hindrance. The purified Switchbodies were ready for validation of antigen‐dependent bioluminescence.

### 2.2 Luminescence of Switchbody is Increased by Addition of Antigen

**Figure 2:**
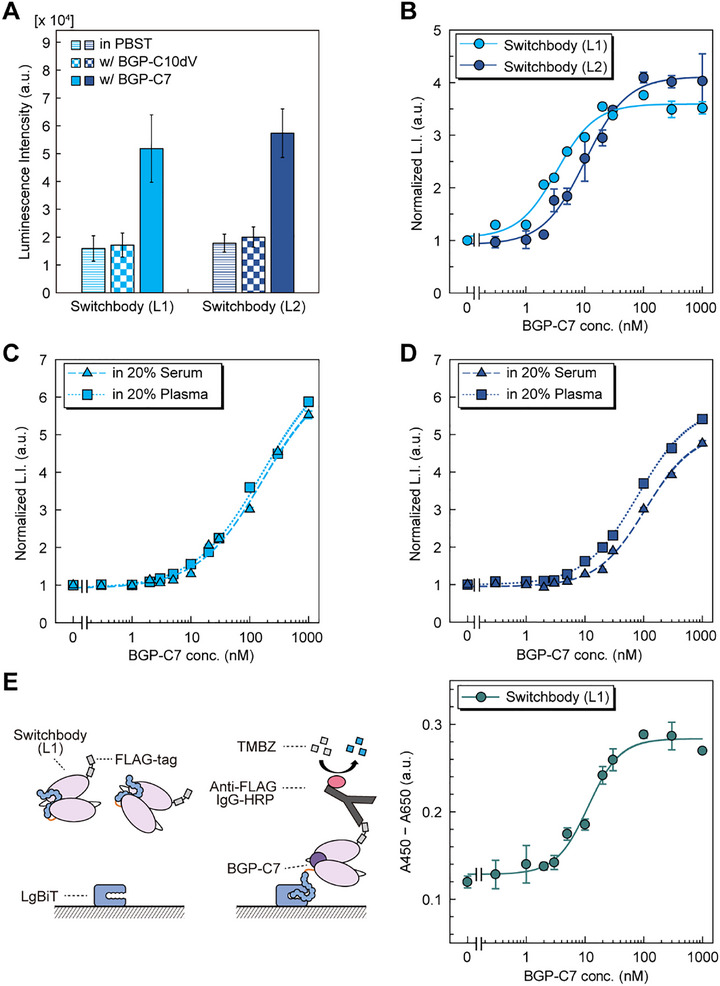
Characterization of Switchbodies. A) Luminescence response of 5 nM Switchbodies with 1 µM BGP‐C7 or BGP‐C10dV. B) Dose‐response curve of 5 nM Switchbodies in PBST (pH 7.4, 0.1% Tween 20). C,D) Dose‐response curve in 20% human serum or 20% human plasma for C) 5 nM Switchbody (L1) and D) 5 nM Switchbody (L2). E) Antigen‐dependent binding activity of Switchbody (L1) to immobilized LgBiT examined by ELISA. Data are shown as mean ± standard deviation of triplicates. L.I.: luminescence intensity. a.u.: arbitrary units.

The luminescence intensity of Switchbodies carrying different linker lengths with LgBiT and substrate was measured with or without antigen (**Figure** **2**A). While both Switchbodies showed >3‐fold increase in luminescence intensity with antigen BGP‐C7 (NH_2_‐RRFYGPV‐COOH), luminescence intensity with BGP‐C10dV (NH_2_‐FQEAYRRFYGP‐COOH), which lacks the C‐terminal valine as a negative control, was comparable to that without antigen. The luminescence intensity of both Switchbodies increased in a dose‐dependent manner (Figure 2B). The maximum responses, EC_50_, and limit of detection (LOD) of the Switchbody (L1) were 3.5‐fold, 3.3 nM, and 0.32 nm, and those of Switchbody (L2) were 4.0‐fold, 10 nM, and 1.2 nM, respectively (Table S2, Supporting Information). These EC_50_ values were slightly higher than the *K*_D_ values of the corresponding Switchbodies. Strikingly, the increase in luminescence intensity of Switchbody (L1) was recognized by a digital camera and even by the naked eye in a dark room (Figure S1, Supporting Information). Furthermore, the luminescence intensity of Switchbodies also increased in a dose‐dependent manner in PBST containing body fluids such as human serum and plasma. However, the EC_50_ value of the Switchbody increased compared to that in PBST alone (Figure 2C,D; Table S2, Supporting Information), consistent with our previous findings,^[^ ^33^, ^34^ ^]^ suggesting a decreased affinity by these complexed media. We consider that the decreased performance may result from interference by components present in serum or plasma with binding between the Switchbody and its antigen, reconstitution of split enzyme, and/or digestion of substrate, necessitating dilution of body fluids. As the working principle of the Switchbody, we considered that upon antigen binding to the scFv region, a trapped HiBiT is released from the scFv and interacts with LgBiT, leading to the reconstitution of NanoLuc and the emission of bioluminescence. To verify the release of HiBiT, we examined the antigen‐dependent binding of Switchbody to immobilized LgBiT by ELISA (Figure 2E). As expected, the absorbance increased in a dose‐dependent manner, suggesting that antigen binding facilitates release of HiBiT from the scFv without cleavage, thereby allowing access to LgBiT.

### 2.3 Understanding HiBiT Behavior in Switchbody by X‐Ray Structural Analysis

**Figure 3:**
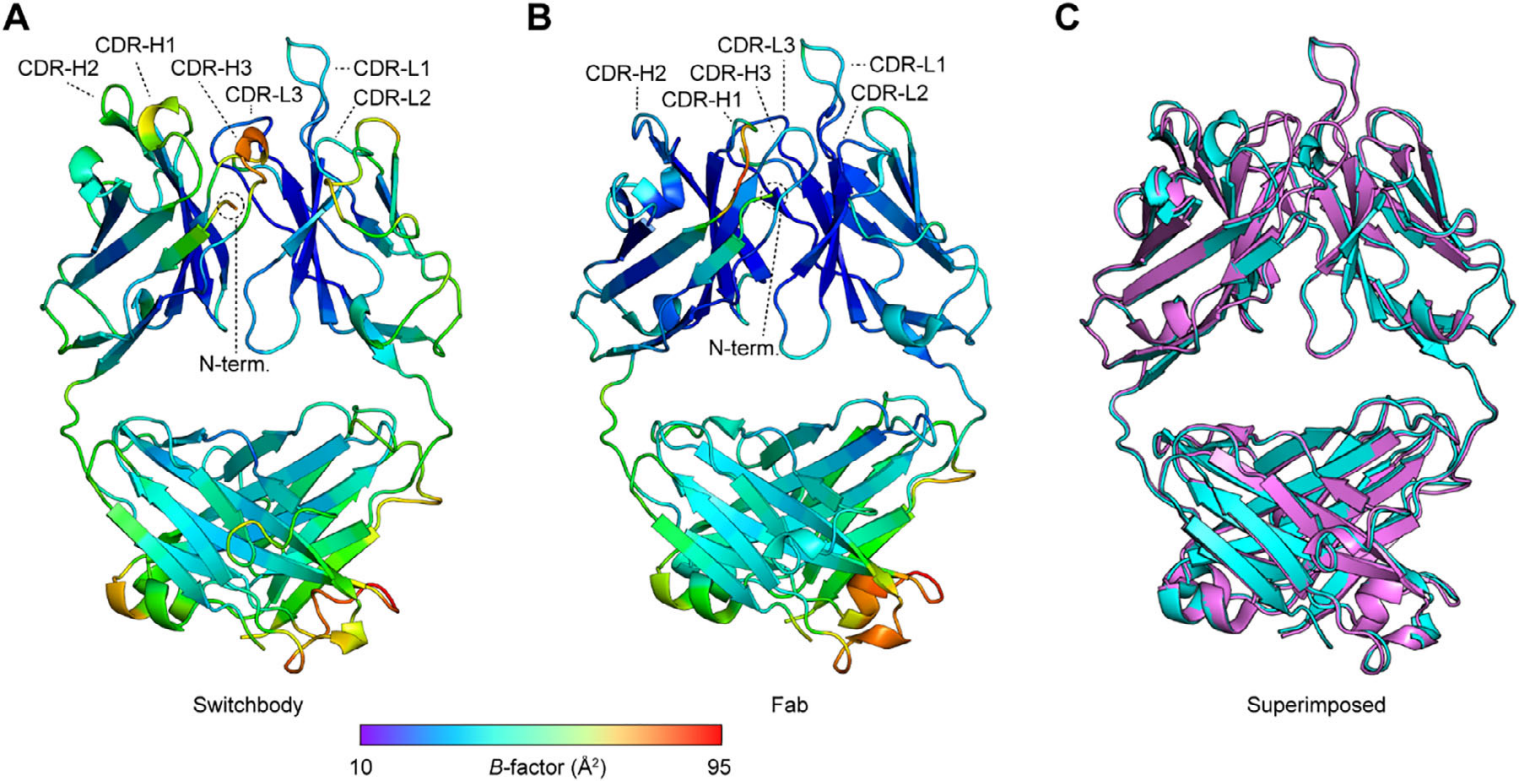
Comparison of X‐ray structures of Fab with and without HiBiT. A,B) Ribbon representation of currently solved Switchbody structure (PDB ID: 9LUK) and previously solved KTM219 Fab structure (PDB ID: 5X5X). Ribbon colors represent the *B*‐factor values as indicated in the scale bar. C) Superimposed structures of Switchbody in cyan and KTM219 Fab in magenta.

We next solved the X‐ray structure of the Switchbody in order to obtain structural insights into the trapping mechanism of HiBiT. The crystal of the Switchbody based on the KTM219 Fab belonged to the orthorhombic space group *P*2_1_2_1_2, with unit cell constants of *a* = 95.84 Å, *b* = 65.91 Å, and *c* = 69.56 Å, and it contained one Switchbody comprising a heavy chain fragment and light chain per asymmetric unit. The structure was refined to 1.95 Å resolution (*R*_work_ = 20.2%, *R*_free_ = 23.8%). All refinement statistics are shown in Table S3 (Supporting Information). While the structure of the Switchbody was solved (PDB ID: 9LUK), the N‐terminal HiBiT region was disordered because its electron density was not visible, suggesting a heterogeneous state of HiBiT in the Switchbody. Compared to the structure of the KTM219 Fab alone, which has been previously solved (PDB ID: 5X5X),^[^ ^35^ ^]^ the atomic fluctuations based on the *B*‐factor in the heavy and light chain complementarity‐determining regions (CDRs) of the Switchbody were relatively larger (**Figure** **3**A,B). Moreover, structural conformations of these heavy chain CDRs differed considerably between Switchbody and Fab alone compared to light chain CDRs and both chains’ framework regions (FRs) (Figure 3C). These results suggest that the HiBiT is mainly located near the heavy chain CDRs and does not tightly bind to a specific site on the antibody in the absence of antigen, similar to the behavior of the fluorescent dye TAMRA in Q‐body.^[^ ^35^, ^36^ ^]^

### 2.4 Analysis of Motility of HiBiT in Switchbody by NMR

**Figure 4:**
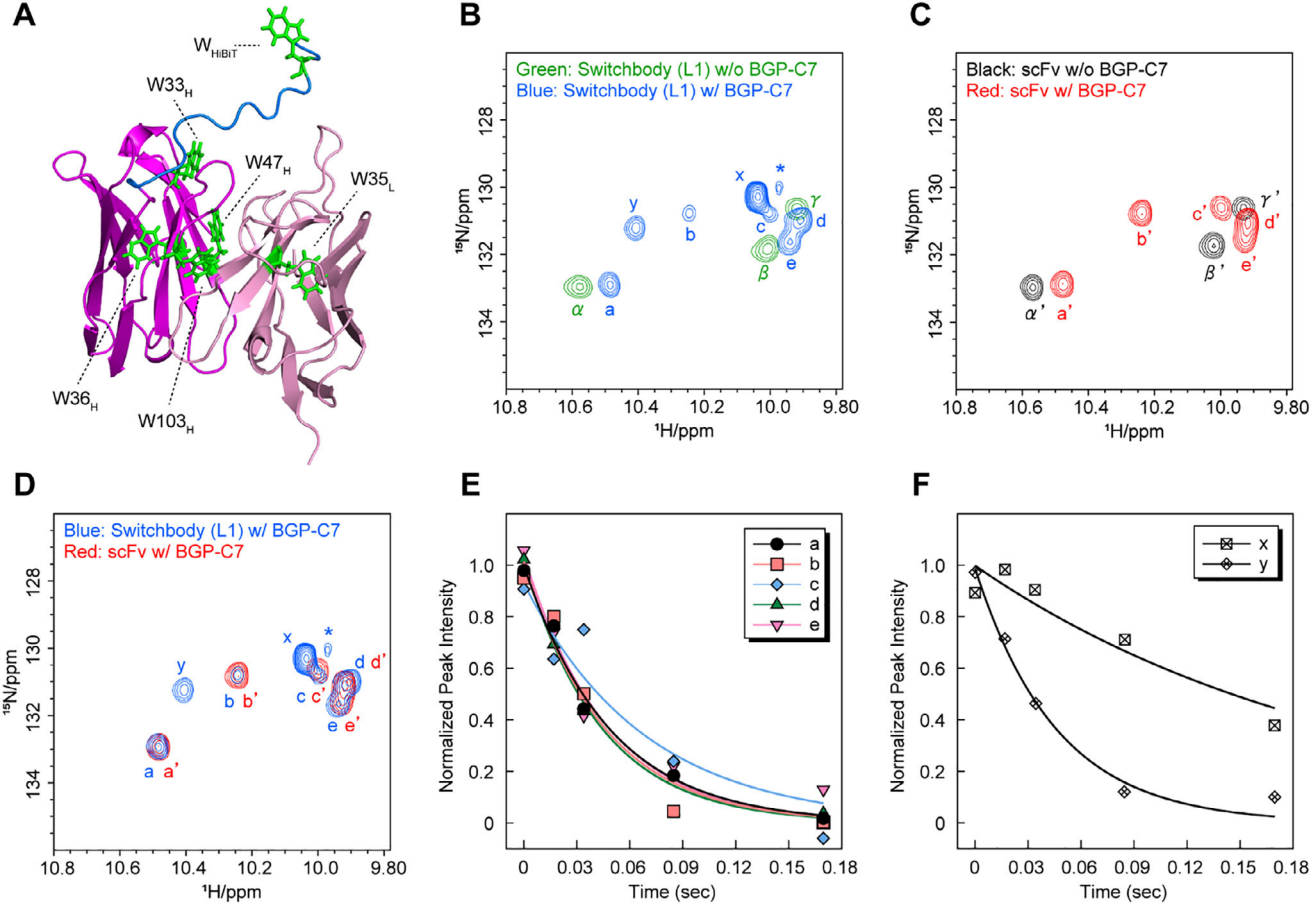
NMR analysis of the dynamic behavior of Switchbody with and without antigen. A) Model structure of Switchbody (L1) generated from the crystal structure (PDB ID: 5X5X). HiBiT is shown in blue, V_H_ in magenta, V_L_ in pink, and Trp in green. B–D) Overlays of ^1^H‐^15^N HSQC spectra: Switchbody (L1) with and without antigen (B), scFv with and without antigen (C), and Switchbody (L1) with antigen and scFv with antigen (D). E,F) Transverse relaxation time *T*_2_ for the Switchbody (L1) with antigen.

We next performed NMR to investigate the dynamics of the Switchbody—and particularly the HiBiT region—in the absence or presence of antigen. We focused on the region around ^1^H 10ppm, ^15^N 130ppm where the signal derived from the NH of the tryptophan (Trp) side chain is generally observed^[^ ^37^, ^38^, ^39^ ^]^ because the HiBiT contains one Trp. The scFv contained in total five Trps, four Trps in V_H_ and one Trp in V_L_ (**Figure** **4**A). When ^1^H‐^15^N heteronuclear single quantum coherence (HSQC) spectra were measured, three signals (α–γ) and seven signals (a–e, x, and y) were observed for the Switchbody (L1) without and with antigen, respectively, on the region around ^1^H 10ppm, ^15^N 130ppm (Figure 4B; Figure S2, Supporting Information). The signal marked with an asterisk is likely a due to aggregation and/or degradation from long‐time analysis since the ^1^H‐^15^N HSQC spectra showed the signal increased in a time‐dependent manner (Figure S3, Supporting Information). Three signals in scFv without antigen (α’–γ’) and five signals in scFv with antigen (a’–e’) were observed at positions similar to signals (α–γ) and (a–e), respectively, in the spectra of the Switchbody (L1) (Figure 4C). We found significant differences in the chemical shift upon adding antigen, suggesting an antigen binding‐induced structural change in the Switchbody (L1) and scFv. From the overlaid spectra of Switchbody (L1) and scFv in the presence of antigen, we observed two signals, x and y, in addition to signals (a–e) and (a’–e’), which were considered to be due to the fusion of HiBiT (Figure 4D). Next, to quantitatively evaluate the dynamics of the HiBiT region of the Switchbody (L1), we measured the transverse relaxation time *T*_2_ for the Switchbody (L1) with antigen and estimated the transverse relaxation rate constant *R*_2_ (s^−1^). The *R*_2_ of signal y was comparable to that of signals (a–e) (Figure 4E,F; Table S4, Supporting Information), while that of signal x was clearly smaller than those of other signals, suggesting that signal y derived from Trp in the scFv region of Switchbody (L1) interacting with HiBiT, and that signal x was other than scFv region or scFv region changed its motility. Considering also that signal x appeared after the addition of antigen, signal x is thought to be derived from a single Trp in HiBiT, which increases motility upon antigen binding.

### 2.5 “Trap and Release” Principle of Switchbody Predicted by MD Simulations

**Figure 5:**
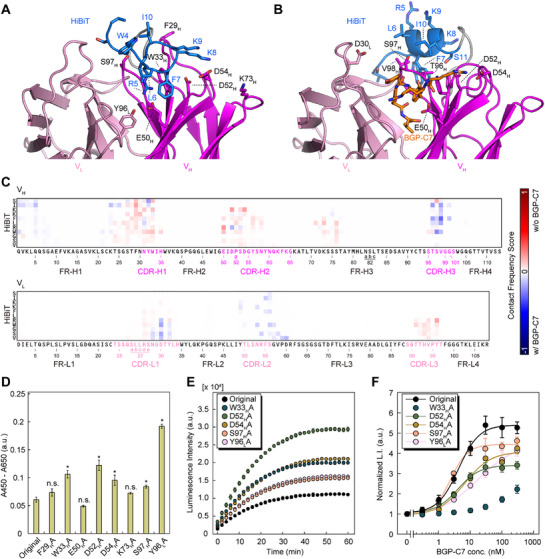
MD simulations and experimental validation of key scFv amino acids for “Trap and Release” of the HiBiT. A,B) Close‐up view of Switchbody, antigen‐binding site generated by MD simulation. HiBiT is shown in blue, V_H_ in magenta, V_L_ in pink, and antigen peptide BGP‐C7 in orange. C) Heatmap derived from MD simulation showing the contact frequency between HiBiT and V_H_ or V_L_ region. FR and CDR regions were defined by Kabat numbering. D) Binding activity of Switchbody mutants to immobilized LgBiT, examined by ELISA. *p < 0.05 by Dunnett's test. n.s.: not significant. E) Time‐course luminescence intensity for Switchbody mutants in the absence of antigen. F) Dose‐response curve of 1 nm Switchbody mutants in PBST (pH 7.4, 0.1% Tween20). Data are shown as mean ± standard deviation of triplicates. L.I.: luminescence intensity. a.u.: arbitrary units.

To further understand the working principle of the Switchbody, we performed molecular dynamics (MD) simulations to predict the interactions between the HiBiT and scFv. Initial model structures of Switchbody based on scFv were generated from the crystal structures with (PDB: 5X5X) and without antigen (PDB: 8XS1),^[^ ^35^ ^]^ and MD simulations were performed for 3.0 µs with five independent runs. For analysis, the last 2.0 µs trajectories were used (total 10 µs for each) (Movies S1 and S2, Supporting Information). Although the HiBiT interacted with heavy chain CDRs near the antigen‐binding site in the absence of antigen, it was moved out of the antigen‐binding site in the presence of antigen (**Figure** **5**A,B). To investigate which amino acid residues of HiBiT and scFv interact with each other, we calculated a contact frequency between the HiBiT and scFv using 10,000 extracted frames of MD simulations (Tables S5 and S6, Supporting Information). The contact frequency was defined as the ratio of the number of frames in which the side chain of each amino acid residue was within 2.5 Å. A difference contact map was created using values obtained by subtracting the contact frequency with antigen from without antigen. HiBiT frequently interacted with CDR‐H1, H2, FR‐H3, and CDR‐L3 in the absence of antigen and with CDR‐H3, CDR‐L1, and CDR‐L2 in the presence of antigen (Figure 5C). These results predict that the position of HiBiT‐scFv interaction is markedly different before and after antigen binding. In addition, by combining these contact maps with the X‐ray structural analysis (Figure 3), we considered that HiBiT interacts with multiple sites in the vicinity of heavy chain CDRs rather than tightly binding to a single specific site on the antibody. To validate these predictions of the scFv amino acids important for trapping HiBiT, we prepared eight alanine (Ala) Switchbody mutants from the top ten most frequently contacted pairs, which are eight positions in the scFv (Figure S4, Supporting Information), and examined the binding of these mutants to immobilized LgBiT by ELISA. In the absence of antigen, the mutants W33_H_A, D52_H_A, D54_H_A, S97_H_A, and Y96_L_A showed significantly higher absorbance signals compared to the original Switchbody (Figure 5D); this suggested that the HiBiT‐scFv interaction was weakened in these Ala mutants, making it easier to reconstitute with LgBiT. As expected, the four mutants showed higher luminescence than the original Switchbody without antigen (Figure 5E), and the luminescence intensity was increased in a dose‐dependent manner (Figure 5F). These results imply that W33_H_, D52_H_, D54_H_, S97_H_A, and Y96_L_ of KTM219 were important for the “Trap and Release” of the HiBiT. In particular, the MD simulations predicted the salt‐bridge interactions between D52_H_ or D54_H_ residues of the scFv and K9 of HiBiT. The D52_H_A or D54_H_A Switchbody mutants showed greater luminescence intensity compared to the original Switchbody in the absence of antigen (Figure 5E), reflecting the weakening of the HiBiT trap in scFv due to destroying the salt‐bridges to Lys by substitution of Asp for Ala. Therefore, one of the key mechanisms for “Trap and Release” of HiBiT would involve the interaction and antigen‐dependent disruption of these salt‐bridges. As interpreted above, given that HiBiT interacts with multiple sites on the antibody, it may be feasible to modulate the intensity and frequency of “Trap and Release” by selectively mutating specific amino acid residues on the antibody for mutation, tailored to the intended application.

### 2.6 Conversion of Label‐Free IgGs into Switchbodies by Antibody Binding Protein

**Figure 6:**
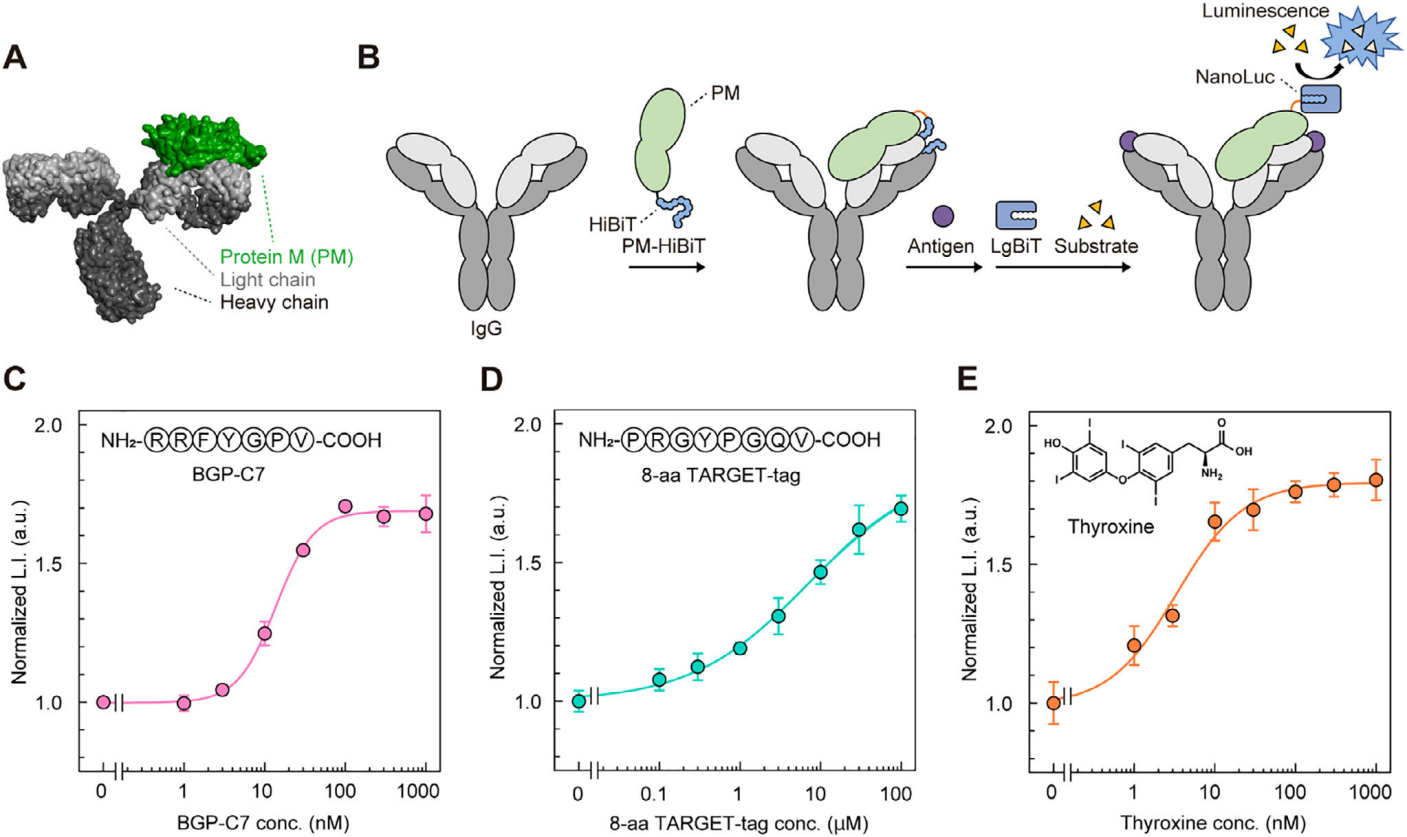
Fabrication of label‐free IgG‐based Switchbodies using antibody‐binding protein. A) Model structure of Protein M/IgG complex generated from their crystal structures (PDB ID: 4NZR and 1IGT). B) Concept of antigen detection using PM‐HiBiT. C–E) Dose‐response curve of complexes of 1 nm PM‐HiBiT and 5 nM IgG in PBST (pH 7.4, 0.1% Tween20): PM‐HiBiT (L3)/KTM219 against BGP‐C7, PM‐HiBiT (L3)/P20.1 against 8‐aa TARGET‐tag, and PM‐HiBiT (L2)/ME.125 against thyroxine. Data are shown as mean ± standard deviation of triplicates. L.I.: luminescence intensity. a.u.: arbitrary units.

We expected that the bioluminescence changes due to the “Trap and Release” of the HiBiT was widely applicable to various antibodies. To efficiently investigate antibodies without genetic manipulation, we prepared a HiBiT‐fused antibody‐binding probe based on Protein M (PM) derived from *Mycoplasma genitalium*.^[^ ^31^ ^]^ The concept is to convert antibodies into bioluminescent Switchbodies, similar to PM Q‐probe system,^[^ ^40^ ^]^ which converts antibodies into fluorescent biosensors by taking advantage of the high‐affinity binding of PM to most antibody light chains from many species (**Figure** **6**A,B). We prepared PM‐HiBiTs carrying linkers of various lengths (L0–3) for optimization (Figure S5A, Supporting Information) and observed the expected bands of PM and PM‐HiBiTs in SDS‐PAGE analysis (Figure S5B, Supporting Information). The binding activity of PM‐HiBiTs to mouse polyclonal IgG was comparable to that of PM alone (Figure S5C, Supporting Information). To optimize the linker length between the PM and HiBiT to obtain a large response, we mixed PM‐HiBiTs (L0–L3) with one model antibody (anti‐BGP IgG KTM219) and two commercially available antibodies (anti‐Thyroxine IgG ME.125 and anti‐TARGET‐tag IgG P20.1) and measured the luminescence intensity with or without antigen. PM‐HiBiT (L2) showed the largest response complexed with anti‐Thyroxine IgG, and PM‐HiBiT (L3) with anti‐BGP IgG and anti‐TARGET‐tag IgG (Figure S5D–F and Table S7, Supporting Information). The luminescence of PM‐HiBiTs/IgGs complexes with antigens was increased in a dose‐dependent manner (Figure 6C–E; Table S8, Supporting Information). However, unlike the low molecular weight antigens described above, which consistently induced an increase in luminescence, the macromolecular antigens produced different responses, with lactoferrin increasing luminescence and C‐reactive protein decreasing it (Figure S5G,H; Table S7, Supporting Information), similar to our turn‐off type antibody‐based biosensor.^[^ ^41^ ^]^ The decrease would be derived from steric hindrance by antigen or interaction with alternative surface from antigen for HiBiT, to inhibit reconstitution of the split enzyme. Taken together, these results suggest that many commercially available antibodies can be converted into immunosensors using PM‐HiBiTs, demonstrating the versatility of “Trap and Release” principle of HiBiT. On the other hand, the complexity arising from using PM‐HiBiTs and bivalent IgGs would affect the quantitative interpretation of an assay. Therefore, PM‐HiBiTs are more appropriate for screening antibodies with potential as Switchbodies, and Switchbodies are preferable for precise quantitative applications. In fluorescent immunosensor Q‐bodies, which we believe utilize the same “Trap and Release” principle, fluorescent dyes such as TAMRA, ATTO520, R6G, etc. have also been shown to be trapped in the antibody via π‐π stacking interaction between xanthene part of dye and side chain of Trp of antibody, and released upon antigen binding.^[^ ^36^, ^42^, ^43^ ^]^ Since antigen‐antibody interactions involve various molecular interactions, including hydrogen bonds, cation‐π interactions, and hydrophobic interactions in addition to salt‐bridges and π‐π stacking interactions,^[^ ^35^, ^44^, ^45^ ^]^ simply placing a short fragment in the vicinity of antigen‐binding site is likely to induce various molecular interactions. In fact, anti‐TARGET‐tag IgG P20.1 was able to induce “Trap and Release” of HiBiT in an antigen‐dependent manner despite the absence of D52_H_ and D54_H_, which are important for salt‐bridges between HiBiT and the anti‐BGP antibody KTM219. Therefore, not only dyes and fused fragments but various other compounds may be trapped in antibodies and released as a switch upon antigen binding.

## 3 Conclusion

Here, we have demonstrated that trap of luciferase‐derived fragment HiBiT near the antigen‐binding site of an antibody and its release in response to an antigen. Based on the “Trap and Release” principle, we successfully developed a luciferase‐based protein switch, bioluminescent Switchbody. In this study, we detected low molecular weight antigens while detection of macromolecular antigens still requires improvement. Future advancements will broaden the utility of bioluminescent Switchbody, enabling the detection of a wide range of targets. Moreover, if the ON/OFF switching state can be precisely regulated by adjusting affinity between antibody and antigen, and split enzyme fragments, the applicability of the Switchbody system will be greatly expanded. “Trap and Release” principle is unique and enables not only the detection and quantification of antigens but also opens new avenues for the precise regulation of cellular physiological functions by antigens, utilizing fragments from split enzymes involved in key processes such as proliferation, differentiation, and metabolism. Furthermore, employing cleavable linkers for drug release^[^ ^41^ ^]^ instead of split enzyme fragments expands the potential for therapeutic applications will facilitate targeted drug release, paving the way for treatments with enhanced target selectivity and minimized side effects.

## Conflict of Interest

T.Y., B.Z., H.U., and T.Kit. received honoraria from HikariQ Health, Inc. for another unrelated project.

## Author Contributions

T.Y., H.U., and T. Kit. conceived the project, designed the experiments. T.Y. and T. Kit. wrote the original manuscript. T.Y. and Y.U. performed plasmid construction, protein preparation, BLI, ELISA, and luminescence measurements. S.Y. and R.A. performed crystallization and X‐ray structural analysis. T.Y., N.T., H.Y., and T. Kig. performed NMR study. T.Y. and M.T. performed MD simulation and its data analysis. B.Z. supported performing experiments. Y.U., M.T., N.T., H.Y., R.A., B.Z., and T. Kig. edited the manuscript. T. Kit. supervised the project. All authors have approved the final version of the manuscript.

## Supporting information



Supporting Information

Supplemental Movie 1

Supplemental Movie 2

Supplemental Data

## Data Availability

Data are available in the Supporting Information file and source data file. The source data file is deposited in the Mendeley Data repository (https://doi.org/10.17632/7d6848crbk.2). The X‐ray crystal structure of Switchbody (L1) have been deposited to the Protein Data Bank (PDB) under accession codes 9LUK. The raw NMR data and MD simulation data are available from the corresponding author upon reasonable request.
