# Supplementary material for: Switchbody, an Antigen‐Responsive Enzyme Switch Based on Antibody and Its Working Principle
Source: Adv Sci (Weinh). 2025 Sep 15;12(44):e08422. doi: 10.1002/advs.202508422 (PMC12667551; doi:10.1002/advs.202508422)
Supplement: Supplementary file 1 — Supporting Information [file ADVS-12-e08422-s002.docx]

Supporting Information for

**Switchbody, an Antigen-Responsive Enzyme Switch Based on Antibody and Its Working Principle**

Takanobu Yasuda, Yoshiyuki Ueno, Masahiko Taguchi, Naoya Tochio, Hiromasa Yagi, Shuma Yazaki, Ryoichi Arai, Bo Zhu, Takanori Kigawa, Hiroshi Ueda^†^ and Tetsuya Kitaguchi*

^†^Deceased 23 December, 2022

*Corresponding author

**Table of Contents**

Experimental Methods

Plasmid construction 3

Protein preparation 3

Bio-Layer Interferometry 4

ELISA 4

Luminescence intensity measurement 5

Dose-response curve fitting 5

Bioluminescence observation in camera 5

Crystallization experiments 5

Data collection, structure determination and refinement 6

Preparation of thermostable scFv for NMR study 6

NMR studies 6

System preparation for MD simulation 7

MD simulation 7

Residue-wise contact map analysis 7

Supplementary Data

**Table S1.** Binding kinetics of scFv and Switchbodies. 8

**Table S2.** Characterization of Switchbodies. 8

**Table S3.** Crystallographic data collection and refinement statistics. 9

**Table S4.** Estimated the apparent transverse relaxation rate constant *R*_2_. 10

**Table S5.** Contact frequency of Switchbody (L1) without antigen. 11

**Table S6.** Contact frequency of Switchbody (L1) with antigen. 11

**Table S7.** Luminescence response of PM-HiBiT/IgG complexes using PM-HiBiT carrying different linker length. 12

**Table S8.** Characterization of PM-HiBiT/IgG complexes. 12

**Table S9.** Half-life of binding activity after 60°C. 12

**Table S10.** Amino acid sequences of scFvs and Switchbodies. 13

**Table S11.** Amino acid sequences of LgBiT. 14

**Table S12.** Amino acid sequences of Protein M and PM-HiBiTs. 14

Figure S1. Bioluminescence images of the 5 nM Switchbody (L1) with various concentrations of BGP-C7 captured using a digital camera. 16

**Figure S2.** Overlay of ^1^H-^15^N HSQC spectra. 17

**Figure S3.** Overlay of ^1^H-^15^N HSQC spectra. 18

**Figure S4.** Preparation of Switchbody mutants. 19

**Figure S5.** Preparation and characterization of PM-HiBiTs. 20

**Figure S6.** Preparation of thermostable scFv for NMR study. 21

**Movie S1.** MD simulation of Switchbody (L1) without antigen. 22

**Movie S2.** MD simulation of Switchbody (L1) with antigen. 22

References 23

**Experimental Methods**

**Plasmid construction**

The DNA fragments encoding the scFvs and Switchbodies were amplified via PCR and inserted into the pSQ vector,^42^ which was digested with NdeI and BamHI. The DNA fragment encoding the LgBiT was amplified via PCR and inserted into the pET-32b(+) vector, which was digested with NcoI and XhoI. The DNA fragments encoding the Protein M (PM) and PM-HiBiTs were amplified via PCR and the products were inserted into the pTrx'st2 vector,^40^ which was digested with NcoI and XhoI. In this study, we used previously reported thermostable PM mutant (PM3mut).^46^ PCR was performed using KOD-Plus-Neo (TOYOBO, #KOD-401). Amplified DNA insertion to each vector was performed using In-Fusion HD Cloning Kit (Takara Bio, #639649). All cloning results were confirmed by Sanger sequencing (Azenta). The amino acid sequences of all the proteins used in this study are listed in supporting information (Tables S10–12).

**Protein preparation**

Expression vector of the protein was transformed into *E. coli* SHuffle T7 Express *lys*Y competent cells (New England Biolabs, #C3030J). Cells were cultured in Luria-Bertani (LB) medium (Difco LB broth, Lennox, BD, #240230) containing 100 µg/mL ampicillin at 30°C to an OD_600_ = 0.4–0.6, then the expression was induced by 0.4 mM isopropyl β-d-thiogalactopyranoside (IPTG) and the culture was incubated for 16 h at 16°C. The cultured cells were collected by centrifugation at 8,000×g for 10 min at 4°C, and the pellet was resuspended in purification buffer (50 mM phosphate, 300 mM NaCl, pH 7.4). They were disrupted by a cell disruptor One shot model (Constant Systems), and the lysate was centrifuged at 8,000×g for 10 min at 4°C to collect the supernatant. The His-tagged protein in the supernatant was purified with TALON Metal Affinity Resin (Takara Bio, #635504) according to the manual and eluted into the elution buffer (50 mM phosphate, 300 mM NaCl, 500 mM Imidazole, pH 7.4). Then, the purified protein was buffer-exchanged to PBS (pH 7.4) using MicroSpin G-25 Columns (Cytiva, #27532501), and glycerol was added at a final concentration of 15% for long-term storage at -30°C. The purified proteins were analyzed by SDS-PAGE, and the concentration was determined by band intensity after Coomassie Brilliant Blue staining using bovine serum albumin as standards on the same gel.

For NMR studies, transformed *E. coli* SHuffle T7 Express *lys*Y competent cells were cultured in 5 mL LB medium containing 50 µg/mL ampicillin overnight at 37°C and subsequently cultured in 9 L M9 minimal medium containing 0.5 g/L ^15^NH_4_Cl for the selective ^15^N-labeling at 37°C to an OD_600_ = 0.5–0.7 using JAR FERMENTOR MBF (TOKYO RIKAKIKAI). Then, expression was induced by 0.4 mM IPTG and the culture was incubated overnight at 16°C. The cultured cells were collected by centrifugation at 6,000 rpm for 10 min at 4°C, and the pellet was resuspended in purification buffer followed by disruption by sonication. After the suspension was centrifuged to collect the supernatant, proteins were purified using TALON Metal Affinity Resin according to the manual and eluted into the elution buffer. Then, the purified protein was buffer-exchanged to the purification buffer using PD-10 column (Cytiva, #17085101). The tag sequence at the N-terminal region of the protein was cleaved by 0.5 µM TEV protease with 1 mM DTT at room temperature overnight and subsequently removed from the protein using TALON Metal Affinity Resin. After buffer-exchange to 20 mM MES buffer (pH 6.0) containing 100 mM NaCl using a PD-10 column, proteins were further purified using a HiTrap SP HP cation exchange chromatography column (Cytiva, #17115201). The collected eluates were concentrated and buffer-exchanged to NMR buffer (20 mM Tris, 300 mM NaCl, 5% D_2_O, pH 7.0) using a Vivaspin 2–10K (Cytiva, #28932247). Finally, approximately 200 µL of 259 µM ^15^N-labeled Switchbody (L1) was obtained. Similarly, approximately 500 µL of 278 µM ^15^N-labeled scFv was obtained from 4.4 L culture using a flask following the same procedure.

**Bio-Layer Interferometry**

Octet K2 System (Sartorius) and streptavidin biosensors (Sartorius, #18-5019) were used for kinetic analyses. Biosensors were hydrated in kinetics buffer (10 mM PB, 150 mM NaCl, 0.002% Tween20, 0.1% BSA) for 10 minutes before assay. 100 nM biotinylated human BGP-C11 peptide (Bio-QEAYRRFYGPV-COOH, LifeTein) in kinetics buffer were incubated with the biosensor until the response signal reached 0.5–0.7 nm then washed in kinetics buffer for 60 seconds. Biosensors with immobilized BGP-C11 peptide were kept hydrated in kinetics buffer for up to a week during experiments. Kinetic assays consisted of a 60 seconds equilibration step in kinetics buffer, a 300 seconds association step, a 600 seconds dissociation step, a 60 seconds regeneration step in regeneration buffer (0.1 M Gly-HCl, 0.1% Tween20, pH 3.0), and a 60 seconds wash step. The assays were performed with four different concentrations of the analytes. For each experiment, the binding curves were fitted to a 1:1 kinetic binding model using the Data Analysis 8.1 HD software (Sartorius) to determine the *k*_on_, *k*_off_, and *K*_D_ values. The data set with χ^2^ < 1.7, R^2^ > 0.996 were used.

**ELISA**

For LgBiT immobilization, 175 nM LgBiT in PBS (pH 7.4) was applied to a clear 96-well microplate (Greiner Bio-One, #655001) and incubated overnight at 4°C. After washing the plate three times with PBST (PBS containing 0.1% Tween20, pH 7.4), PBS (pH 7.4) containing 20% ImmunoBlock (KAC, #CTKN001), 1 nM Switchbody, and 0.1 µg/mL anti-FLAG IgG-HRP were successively added to the wells. Each reagent was incubated individually for 2 hours, 20 minutes, and 30 minutes, respectively. After each incubation step, the wells were washed three times with PBST. After each incubation step, the wells were washed three times with PBST. Subsequently, a substrate solution (100 mM sodium acetate, 100 µg/mL TMBZ, 0.03% H_2_O_2_, pH 6.0) was applied and incubated for few minutes. The reaction was stopped with 10% H_2_SO_4_, and the absorbance was measured at 450 nm with a reference at 650 nm using a microplate reader SH-1000 (Corona Electric).

For mouse IgG immobilization, 10 µg/mL mouse IgG (FUJIFILM Wako, #140-09511) in PBS (pH 7.4) was applied to a clear 96-well microplate and incubated overnight at 4°C. After washing the plate three times with PBST, PBS (pH 7.4) containing 20% ImmunoBlock, 5 nM Protien M or PM-HiBiT, and 1/5000 diluted StrepTactin-HRP (Bio-Rad, #1610380) were successively added to the wells. Each reagent was incubated individually for 2 hours, 1 hour, and 30 minutes, respectively. After each incubation step, the wells were washed three times with PBST. The subsequent procedure was the same as described above.

**Luminescence intensity measurement**

Purified LgBiT and NanoGlo substrate (Promega, #N1110) were mixed at a final concentration of 1–5 nM and a 2000-fold dilution, respectively, and were applied to a white 96-well microplate (Greiner Bio-One, #675075). Separately, 1–5 nM Switchbody, pre-incubated with or without the antigen peptide (LifeTein) in PBST (pH 7.4) for 30 minutes at room temperature, was also applied to the white microplate. Immediately afterward luminescence intensity at 460 nm with a bandwidth of 30 nm was measured using a CLARIOstar (BMG LABTECH).

For measurements under crude conditions, either human serum or human plasma was mixed to a final concentration of 20% in PBST during sample preparation. For PM-HiBiT assay, 1 nM PM or PM-HiBiT was incubated with 5 nM of each IgG: anti-BGP IgG (clone KTM219, lab-made), anti-TARGET-tag IgG (clone P20.1, FUJIFILM Wako, #016-25481), anti-Thyroxine IgG (clone ME.125, Abnova, #MAB4752), anti-Lactoferrin IgG (clone 1A1, HyTest, #4L2), anti-C-reactive protein IgG (clone 160.10G10, Bio-Rad, #MCA5880G). The incubation was performed in PBST (pH 7.4) for 40 minutes at room temperature before measurement.

**Dose-response curve fitting**

Dose-response curves were fitted to a four-parameter logistic equation as below using ImageJ 1.54i software (Wayne Rasband).

$$y=d+\frac{a-d}{1+\left( \frac{x}{c} \right)^{b}}$$

The limit of detection was calculated as the concentration corresponding to the mean blank value plus three times its standard deviation.

**Bioluminescence observation in camera**

To observe and capture images of luminescence, 5 nM Switchbody (L1), 0.3, 2, 5, 20, 100, 1,000 nM BGP-C7, 5 nM LgBiT, and NanoGlo substrate at a final dilution of 1000-fold in PBST were added to a white 96-well microplate. Images were taken using a digital camera α7S (Sony) set at ISO 8000, F1/2, with five seconds exposure in a dark room.

**Crystallization experiments**

The Fab-based Switchbody was purified using immobilized metal affinity chromatography with TALON Metal Affinity Resin, anion exchange chromatography with a RESOURCE Q 1 mL column (Cytiva, #17117701), and size-exclusion chromatography with a Superdex 75 Increase 10/300 GL column (Cytiva, #29148721), as previously described^35^. The 10 mg/mL protein was crystallized at 20°C using the hanging drop vapor diffusion method. The 1 µL Switchbody was mixed with the same volume of reservoir solution (0.1 M Bis-Tris, pH 6.0, 22% w/v polyethylene glycol monomethyl ether 2000).

**Data collection, structure determination and refinement**

X-ray diffraction data was collected at KEK Photon Factory (PF) Structural Biology Beamline BL-5A or AR-NW12A at 95 K with reservoir solution added to 25% w/v PEG 400 or 25% w/v glycerol as a cryoprotectant. Diffraction data was processed with the program XDS^47^ and AIMLESS^48^. The structure was solved by molecular replacement method using Phaser with a model structure of anti-osteocalcin antibody KTM219 (PDB ID: 5X5X)^35^. The structural model was corrected with the program COOT^49^ and was refined with the program REFMAC5^50^ in the CCP4 suite^51^. The quality of the model was inspected by the programs PROCHECK^52^, RAMPAGE^53^, and MolProbity^54^. All data collection and refinement statistics are shown in Table S3. The atomic coordinates and the structure factors have been deposited in the Protein Data Bank (PDB) with the accession codes 9LUK. The graphic figures were created using the program PyMOL (Schrödinger).

**Preparation of thermostable scFv for NMR study**

Since high-concentration samples (> 200 µM) were required for NMR structural analysis, tyrosine at position 27 of the heavy chain in the scFv was mutated to serine to improve thermal stability. Initially, F11_H_, Y27_H_, F29_H_, and L9_L_ were selected as candidates for thermostable mutations based on computational predictions of sidechain solvent accessibility and hydrophobicity of the scFv, using Discovery Studio Visualizer v18.1 (BIOVIA) (Figure S6A, B). These selected hydrophobic amino acids were mutated to hydrophilic amino acids, either serine or threonine, and the antigen-binding activity of the mutants was evaluated by ELISA (Figure S6C). To assess the thermal stability of each mutant, the binding activity of the mutants to immobilized antigen after incubation at 60°C for various time was examined by ELISA. Among the wild-type and mutants, Y27_H_S mutant exhibited the longest half-time of binding activity after incubation at 60°C (Figure S6D, Table S9). Consequently, we determined to use the Y27_H_S mutant for NMR study, MD simulation and subsequent luminescence intensity measurements.

**NMR studies**

The ^1^H-^15^N HSQC spectra were measured using an Avance III 700-MHz spectrometer (Bruker Biospin) at 298 K. The ^15^N-labeled protein sample was dissolved in an NMR buffer (20 mM Tris, 300 mM NaCl, 5% D_2_O, pH 7.0) at a concentration of 200 µM, either with or without 300 µM antigen BGP-C7. The interscan delay was set to 1.7 seconds, the number of data integrations was 32, and the number of data points were 1,024 pts [^1^H] and 128 pts [^15^N]. For the transverse relaxation time *T*_2_ measurements, the delay intervals were set to 0, 17.0, 33.9, 84.8, and 169.6 ms. The apparent transverse relaxation rate constant *R*_2_ was estimated by fitting peak intensities using Monte Carlo method (N = 128) as a function of above 5 relaxation delay intervals based on the following equation.

$$I_{t}=I_{0}\cdot e^{-t{\cdot R}_{2}}$$

All NMR spectra were processed using TopSpin 3.6.2 software (Bruker Biospin).

**System preparation for MD simulation**

The initial simulation structures were constructed using X-ray crystallographic structures of Fab fragment (KTM219) with and without antigen (PDB ID: 8XS1 and 5X5X, respectively) to match protein sequences used in the NMR experiment. Crystallographic water molecules within 5.0 Å from the extracted protein region are retained. Structures of HiBiT, linker between HiBiT and V_H_ region, as well as one between V_H_ and V_L_ regions are modelled by MODELLER^55^. Missing hydrogen atoms were added to the protein with the LEaP module of AMBER20. The simulation systems were solvated with TIP3P water molecules^56^ with a minimum distance of 15.0 Å between the protein and the box edges, and Na^+^ and Cl^-^ ions were added to neutralize the systems and match condition in the NMR experiment (300 mM NaCl). The force field parameter set of AMBER ff14SB^57^ and that reported in reference^58^ were employed for the force fields of the protein, Na^+^ and Cl^-^ ions, respectively. The total number of atoms in the box were 80,266 and 60,569 atoms for system with and without antigen.

**MD simulation**

GPU modules of the AMBER20 program package^59^ were employed. The simulation systems were first subject to energy minimization under harmonic restraints to heavy atoms of the protein with a force constant of 5.0 kcal/(mol·Å^2^). The structures were further energetically minimized under harmonic restraints to C_α_ atoms of the protein with a force constant of 5.0 kcal/(mol·Å^2^). Next, the systems were heated from 0 K to 298 K for 300 ps in NVT condition under harmonic restraints of C_α_ atoms of the protein with a force constant of 5.0 kcal/(mol·Å^2^). After the heating, the systems were gradually relaxed in NPT condition with decreasing the harmonic restraints to C_α_ atoms of the protein with a force constant of 5.0, 3.0, 2.0, 1.0, 0.5, 0.3, 0.2, and 0.1 kcal/(mol·Å^2^) for every 1.0 ns. Then, five independent production runs at 298 K for 3.0 µs in NPT condition were performed. Long-range electrostatic interactions were calculated using the particle mesh Ewald method^60^. Temperature and pressure were controlled with Langevin bath (collision frequency is 2.0 ps^-1^) and Berendsen’s methods^61^. Nonbonded interactions were cut off at 8.0 Å, and bond lengths including hydrogen atoms were constrained by the SHAKE/RATTLE method^62,63^. The time step for integration was set to 2.0 fs. For analysis, the last 2.0 µs trajectories were used. The total MD trajectory for analysis is 10 µs for each system. Molecular figures were generated using the PyMOL Molecular Graphics System (Schrödinger). Molecular movies were generated using the VMD^64^.

**Residue-wise contact map analysis**

Contact map analysis was performed by MDTraj^65^. Residue-wise intra-contact was counted if any of side-chain atoms from a pair of residues is less than 2.5 Å. Then, differences of contacts were calculated by subtracting contacts of the system without antigen from those of the system with antigen.

**Supplementary Data**

**Table S1.** Binding kinetics of scFv and Switchbodies.

| Sample | *K*_D_ (M) | *k*_on_ (M^-1^s^-1^) | *k*_off_ (s^-1^) |
| --- | --- | --- | --- |
| scFv | 0.81×10^-9^ | 1.3×10^5^ | 1.0×10^-4^ |
| Switchbody (L1) | 1.6×10^-9^ | 0.61×10^5^ | 1.0×10^-4^ |
| Switchbody (L2) | 2.3×10^-9^ | 0.51×10^5^ | 1.2×10^-4^ |

**Table S2.** Characterization of Switchbodies.

| Sample | Buffer | Response (-fold) | EC_50_ (nM) | LOD (nM) |
| --- | --- | --- | --- | --- |
| Switchbody (L1) | PBST | 3.5 | 3.3 | 0.32 |
|  | 20% Serum | 5.5 | 165 | 1.3 |
|  | 20% Plasma | 5.9 | 163 | 2.7 |
| Switchbody (L2) | PBST | 4.0 | 10 | 1.2 |
|  | 20% Serum | 4.8 | 107 | 4.6 |
|  | 20% Plasma | 5.4 | 80 | 1.6 |

Data are shown as mean of triplicates.

**Table S3.** Crystallographic data collection and refinement statistics.

| *Data collection* | |
| --- | --- |
| Space group | *P*2_1_2_1_2 |
| Unit-cell parameters (Å) | *a* = 95.84  *b* = 65.91  *c* = 69.56 |
| X-ray source | PF BL-5A |
| Wavelength (Å) | 1.00 |
| Resolution (Å) | 47.92–1.95 (2.00–1.95) |
| Unique reflections | 32857 (2290) |
| Average redundancy | 13.1 (12.7) |
| Completeness (%) | 100.0 (100.0) |
| *I* / σ(*I*) | 23.9 (2.2) |
| *R*_meas_ (%) | 7.6 (130.8) |
| CC_1/2_ | 1.000 (0.850) |
| *Refinement* | |
| Resolution (Å) | 47.92–1.95 |
| No. of reflections | 31276 |
| No. of protein atoms | 3274 |
| No. of water atoms | 273 |
| No. of other atoms | 6 |
| *R*_work_ (%) | 20.2 |
| *R*_free_ (%) | 23.8 |
| RMSD bond length (Å) | 0.012 |
| RMSD bond angles (˚) | 1.47 |
| Average *B*-factor (Å^2^) | 41.3 |
| *Ramachandran plot* | |
| In preferred regions (%) | 97.4 |
| In allowed regions (%) | 2.6 |
| Outliers (%) | 0 |
| PDB ID | 9LUK |

All numbers in parentheses represent the last outer shell statistics.

*R*_free_ is calculated for 4.7% of randomly selected reflections excluded from refinement.

Ramachandran plot analyses of the models were performed using RAMPAGE.

**Table S4.** Estimated the apparent transverse relaxation rate constant *R*_2_.

| Signal | *R*_2_ (s^-1^) |
| --- | --- |
| a | 20 ± 3.7 |
| b | 19 ± 5.1 |
| c | 17 ± 4.6 |
| d | 22 ± 2.8 |
| e | 22 ± 6.0 |
| x | 4.9 ± 0.63 |
| y | 20 ± 6.9 |

Data are shown as mean ± standard deviation of curve fitting using Monte Carlo method (N = 128).

**Table S5.** Contact frequency of Switchbody (L1) without antigen.

| Rank | Residue pair | | Contact frequency (%) |
| --- | --- | --- | --- |
|  | HiBiT | scFv |  |
| 1 | R5 | E50_H_ | 20 |
| 2 | K9 | D52_H_ | 19 |
| 3 | K9 | D54_H_ | 19 |
| 4 | I10 | F29_H_ | 18 |
| 5 | R5 | Y96_L_ | 18 |
| 6 | S11 | S97_H_ | 16 |
| 7 | S11 | W33_H_ | 13 |
| 8 | K9 | W33_H_ | 12 |
| 9 | W4 | E50_H_ | 12 |
| 10 | L6 | K73_H_ | 11 |

**Table S6.** Contact frequency of Switchbody (L1) with antigen.

| Rank | Residue pair | | Contact frequency (%) |
| --- | --- | --- | --- |
|  | HiBiT | scFv |  |
| 1 | F7 | V98_H_ | 30 |
| 2 | W4 | S97_H_ | 23 |
| 3 | S11 | Y32_H_ | 19 |
| 4 | I10 | T96_H_ | 19 |
| 5 | I10 | V98_H_ | 19 |
| 6 | W4 | D30_L_ | 18 |
| 7 | F7 | T96_H_ | 17 |
| 8 | S2 | D30_L_ | 17 |
| 9 | L6 | V98_H_ | 16 |
| 10 | R5 | D52_H_ | 15 |

**Table S7.** Luminescence response of PM-HiBiT/IgG complexes using PM-HiBiT carrying different linker length.

| IgG | Response (-fold) | | | |
| --- | --- | --- | --- | --- |
|  | L0 | L1 | L2 | L3 |
| KTM219 | 1.1 | 1.2 | 1.6 | 1.7 |
| P20.1 | 1.4 | 1.3 | 1.4 | 1.4 |
| ME.125 | 1.4 | 1.4 | 2.0 | 1.3 |
| 1A1 | 1.7 | 2.2 | 2.1 | 2.0 |
| 160.10G10 | 0.47 | 0.57 | 0.55 | 0.67 |

Data are shown as a single measurement only for screening purpose.

**Table S8.** Characterization of PM-HiBiT/IgG complexes.

| PM-HiBiT/IgG | Response (-fold) | EC_50_ | LOD |
| --- | --- | --- | --- |
| L3/KTM219 | 1.7 | 14 nM | 1.9 nM |
| L3/P20.1 | 1.7 | 6.9 µM | 0.33 µM |
| L2/ME.125 | 1.8 | 2.8 nM | 1.5 nM |

Data are shown as mean of triplicates.

**Table S9.** Half-life of binding activity after 60°C.

| Variant | T_1/2_ (h) |
| --- | --- |
| WT | 2.2 |
| Y27_H_S | 3.8 |
| Y27_H_T | 2.7 |
| F29_H_S | 2.7 |
| L9_L_S | 1.7 |
| L9_L_T | 1.5 |

**Table S10.** Amino acid sequences of scFvs and Switchbodies.

| Name | Sequence |
| --- | --- |
| scFv | MSKIKQVKLQQSGAEFVKAGASVKLSCKTSGYTFNNYWIHWVKQSPGQGLEWIGEIDPSDGYSNYNQKFKGKATLTVDKSSSTAYMHLNSLTSEDSAVYYCTSSTSVGGSWGQGTTVTVSSGGGGSGGGGSGGGGSDIELTQSPLSLPVSLGDQASISCTSSQSLLHSNGDTYLHWYLQKPGQSPKLLIYTLSNRFSGVPDRFSGSGSGTDFTLKISRVEAADLGIYFCSQTTHVPYTFGGGTKLEIKRGGGSHHHHHHGGSDYKDDDDK* |
| Switchbody (L1) | MSVSGWRLFKKISGGGSTGQVKLQQSGAEFVKAGASVKLSCKTSGYTFNNYWIHWVKQSPGQGLEWIGEIDPSDGYSNYNQKFKGKATLTVDKSSSTAYMHLNSLTSEDSAVYYCTSSTSVGGSWGQGTTVTVSSGGGGSGGGGSGGGGSDIELTQSPLSLPVSLGDQASISCTSSQSLLHSNGDTYLHWYLQKPGQSPKLLIYTLSNRFSGVPDRFSGSGSGTDFTLKISRVEAADLGIYFCSQTTHVPYTFGGGTKLEIKRGGGSHHHHHHGGSDYKDDDDK* |
| Switchbody (L2) | MSVSGWRLFKKISGGGSGGGSTGQVKLQQSGAEFVKAGASVKLSCKTSGYTFNNYWIHWVKQSPGQGLEWIGEIDPSDGYSNYNQKFKGKATLTVDKSSSTAYMHLNSLTSEDSAVYYCTSSTSVGGSWGQGTTVTVSSGGGGSGGGGSGGGGSDIELTQSPLSLPVSLGDQASISCTSSQSLLHSNGDTYLHWYLQKPGQSPKLLIYTLSNRFSGVPDRFSGSGSGTDFTLKISRVEAADLGIYFCSQTTHVPYTFGGGTKLEIKRGGGSHHHHHHGGSDYKDDDDK* |
| scFv (Y27_H_S)  for NMR | MSKIKHHHHHHSSGENLYFQGGGGSTGQVKLQQSGAEFVKAGASVKLSCKTSGSTFNNYWIHWVKQSPGQGLEWIGEIDPSDGYSNYNQKFKGKATLTVDKSSSTAYMHLNSLTSEDSAVYYCTSSTSVGGSWGQGTTVTVSSGGGGSGGGGSGGGGSDIELTQSPLSLPVSLGDQASISCTSSQSLLHSNGDTYLHWYLQKPGQSPKLLIYTLSNRFSGVPDRFSGSGSGTDFTLKISRVEAADLGIYFCSQTTHVPYTFGGGTKLEIKR* |
| Switchbody (L1, Y27_H_S)  for NMR | MSKIKHHHHHHSSGENLYFQGVSGWRLFKKISGGGSTGQVKLQQSGAEFVKAGASVKLSCKTSGSTFNNYWIHWVKQSPGQGLEWIGEIDPSDGYSNYNQKFKGKATLTVDKSSSTAYMHLNSLTSEDSAVYYCTSSTSVGGSWGQGTTVTVSSGGGGSGGGGSGGGGSDIELTQSPLSLPVSLGDQASISCTSSQSLLHSNGDTYLHWYLQKPGQSPKLLIYTLSNRFSGVPDRFSGSGSGTDFTLKISRVEAADLGIYFCSQTTHVPYTFGGGTKLEIKR* |

VH and VL; HiBiT; TEV site; His-tag and FLAG-tag

**Table S11.** Amino acid sequences of LgBiT.

| Name | Sequence |
| --- | --- |
| LgBiT | MVFTLEDFVGDWEQTAAYNLDQVLEQGGVSSLLQNLAVSVTPIQRIVRSGENALKIDIHVIIPYEGLSADQMAQIEEVFKVVYPVDDHHFKVILPYGTLVIDGVTPNMLNYFGRPYEGIAVFDGKKITVTGTLWNGNKIIDERLITPDGSMLFRVTINSLEHHHHHH* |

LgBiT; His-tag

**Table S12.** Amino acid sequences of Protein M and PM-HiBiTs.

| Name | Sequence |
| --- | --- |
| Protein M | MGNDGSYQSEIDLSGGANFREKFRNFANELSEAITNSPKGSDRPVPKTEISGLIKTGDNFITPSFKAGYYDHVASDGSLLSYYQSTEYFNNRVLMPILQTTNGTLMANNRGYDDVFRQVPSFSGWSNTKATTVSTSNNLTYDKWTYYAAKGSPLYDSYPNHSFEDVKTLAIDAKDISALKTTIDSEKPTYLIIRGLSGNGSQLNELQLPESVKKVSLYGDYTGVNVAKQIFANVVELEFYSTSKANSFGFNPLVLGSKTNVIYDLFASKPFTHIDLTQVTLQNSDNSAIDANKLKQAVGDIYNYRRFERQFQGYFAGGYIDKYLVKNVNTNKDSDDDLVYRSLKELNLHLEEAYREGDNTYYRVNENYYPGASIYENERASRDSEFQNEILKR* |
| PM-HiBiT (L0) | MGNDGSYQSEIDLSGGANFREKFRNFANELSEAITNSPKGSDRPVPKTEISGLIKTGDNFITPSFKAGYYDHVASDGSLLSYYQSTEYFNNRVLMPILQTTNGTLMANNRGYDDVFRQVPSFSGWSNTKATTVSTSNNLTYDKWTYYAAKGSPLYDSYPNHSFEDVKTLAIDAKDISALKTTIDSEKPTYLIIRGLSGNGSQLNELQLPESVKKVSLYGDYTGVNVAKQIFANVVELEFYSTSKANSFGFNPLVLGSKTNVIYDLFASKPFTHIDLTQVTLQNSDNSAIDANKLKQAVGDIYNYRRFERQFQGYFAGGYIDKYLVKNVNTNKDSDDDLVYRSLKELNLHLEEAYREGDNTYYRVNENYYPGASIYENERASRDSEFQNEILKRAASVSGWRLFKKIS* |
| PM-HiBiT (L1) | MGNDGSYQSEIDLSGGANFREKFRNFANELSEAITNSPKGSDRPVPKTEISGLIKTGDNFITPSFKAGYYDHVASDGSLLSYYQSTEYFNNRVLMPILQTTNGTLMANNRGYDDVFRQVPSFSGWSNTKATTVSTSNNLTYDKWTYYAAKGSPLYDSYPNHSFEDVKTLAIDAKDISALKTTIDSEKPTYLIIRGLSGNGSQLNELQLPESVKKVSLYGDYTGVNVAKQIFANVVELEFYSTSKANSFGFNPLVLGSKTNVIYDLFASKPFTHIDLTQVTLQNSDNSAIDANKLKQAVGDIYNYRRFERQFQGYFAGGYIDKYLVKNVNTNKDSDDDLVYRSLKELNLHLEEAYREGDNTYYRVNENYYPGASIYENERASRDSEFQNEILKRAASGGGSVSGWRLFKKIS* |
| PM-HiBiT (L2) | MGNDGSYQSEIDLSGGANFREKFRNFANELSEAITNSPKGSDRPVPKTEISGLIKTGDNFITPSFKAGYYDHVASDGSLLSYYQSTEYFNNRVLMPILQTTNGTLMANNRGYDDVFRQVPSFSGWSNTKATTVSTSNNLTYDKWTYYAAKGSPLYDSYPNHSFEDVKTLAIDAKDISALKTTIDSEKPTYLIIRGLSGNGSQLNELQLPESVKKVSLYGDYTGVNVAKQIFANVVELEFYSTSKANSFGFNPLVLGSKTNVIYDLFASKPFTHIDLTQVTLQNSDNSAIDANKLKQAVGDIYNYRRFERQFQGYFAGGYIDKYLVKNVNTNKDSDDDLVYRSLKELNLHLEEAYREGDNTYYRVNENYYPGASIYENERASRDSEFQNEILKRAASGGGSGGGSVSGWRLFKKIS* |
| PM-HiBiT (L3) | MGNDGSYQSEIDLSGGANFREKFRNFANELSEAITNSPKGSDRPVPKTEISGLIKTGDNFITPSFKAGYYDHVASDGSLLSYYQSTEYFNNRVLMPILQTTNGTLMANNRGYDDVFRQVPSFSGWSNTKATTVSTSNNLTYDKWTYYAAKGSPLYDSYPNHSFEDVKTLAIDAKDISALKTTIDSEKPTYLIIRGLSGNGSQLNELQLPESVKKVSLYGDYTGVNVAKQIFANVVELEFYSTSKANSFGFNPLVLGSKTNVIYDLFASKPFTHIDLTQVTLQNSDNSAIDANKLKQAVGDIYNYRRFERQFQGYFAGGYIDKYLVKNVNTNKDSDDDLVYRSLKELNLHLEEAYREGDNTYYRVNENYYPGASIYENERASRDSEFQNEILKRAASGGGSGGGSGGGSVSGWRLFKKIS* |

Protein M; HiBiT; His-tag; Linker


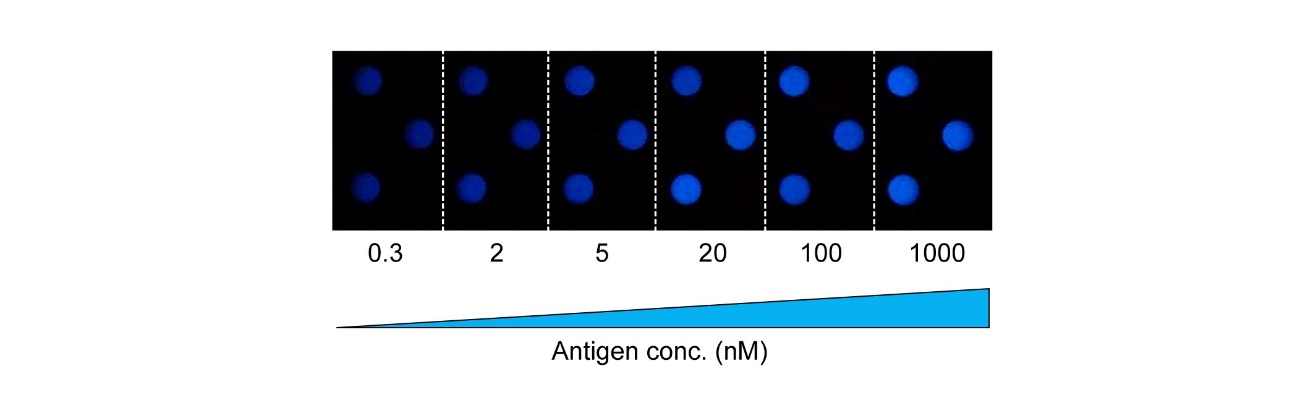


Figure S1. Bioluminescence images of the 5 nM Switchbody (L1) with various concentrations of BGP-C7 captured using a digital camera.


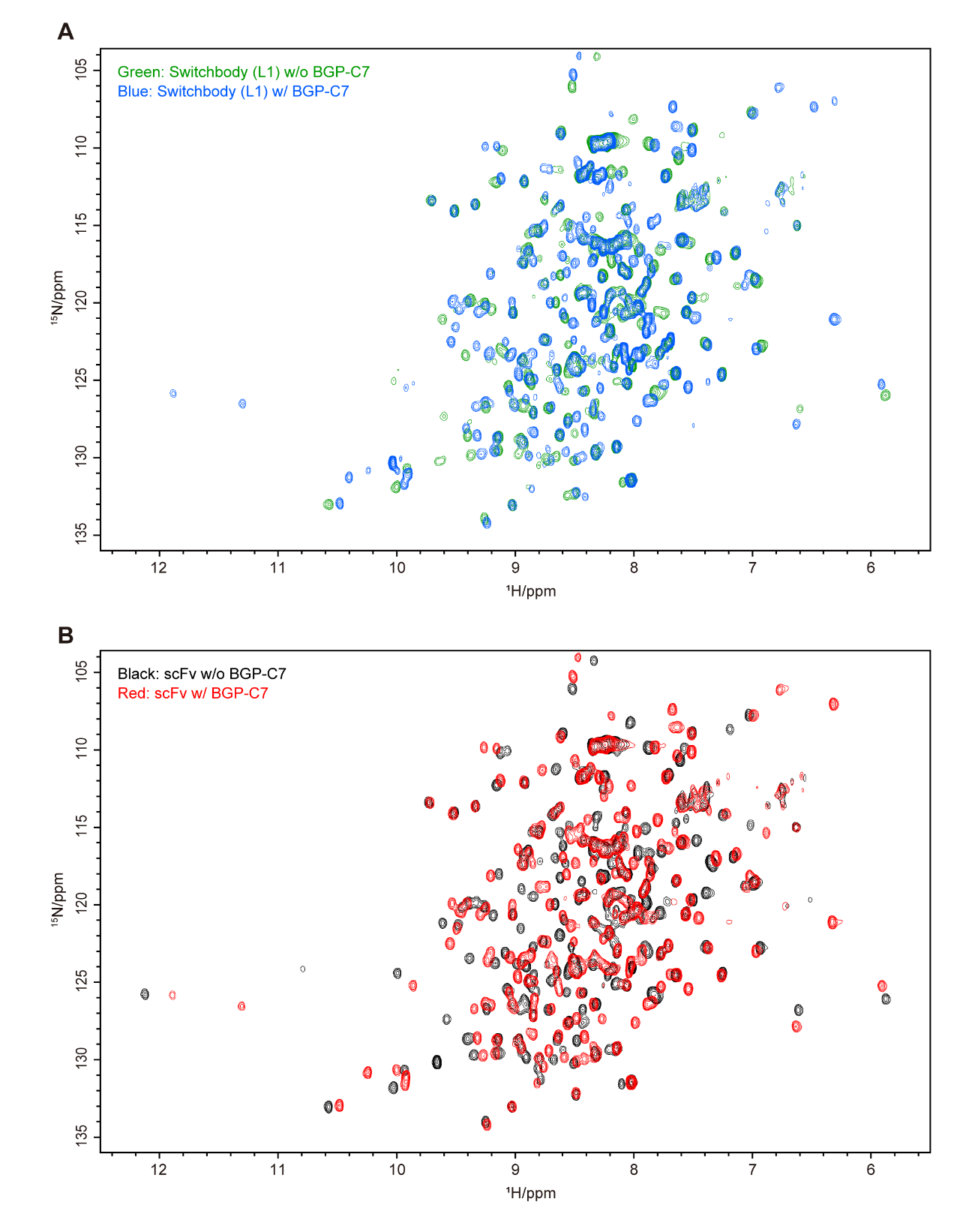


Figure S2. Overlay of ^1^H-^15^N HSQC spectra. (A) Switchbody (L1) with and without antigen. (B) scFv with and without antigen.


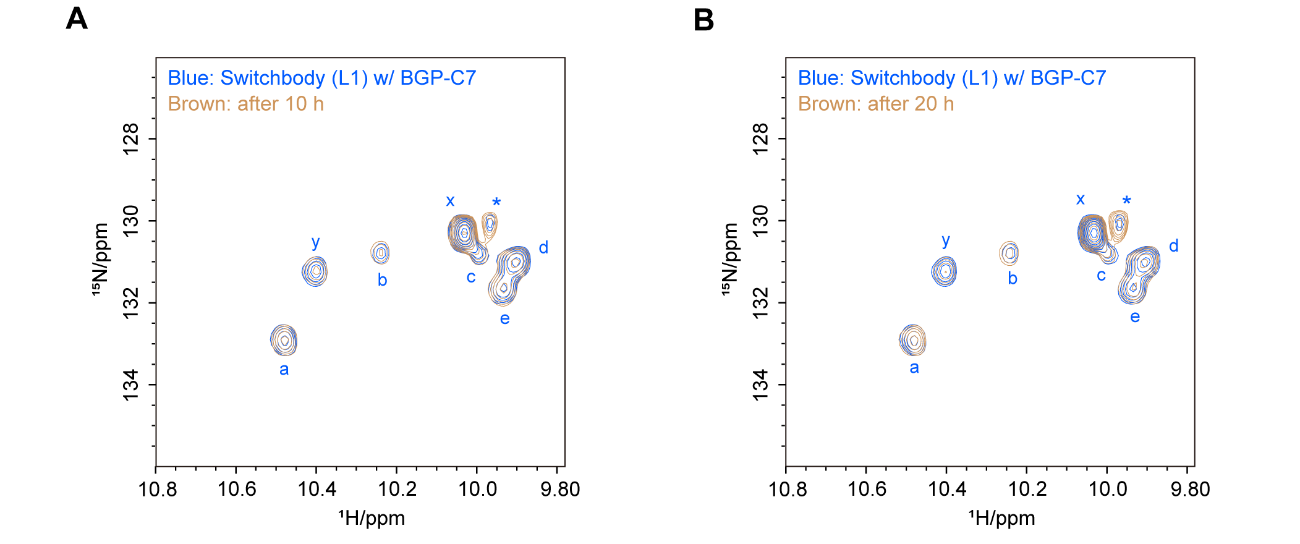


Figure S3. Overlay of ^1^H-^15^N HSQC spectra. (A) Switchbody (L1) with antigen and after 10 hours. (B) Switchbody (L1) with antigen and after 20 hours.


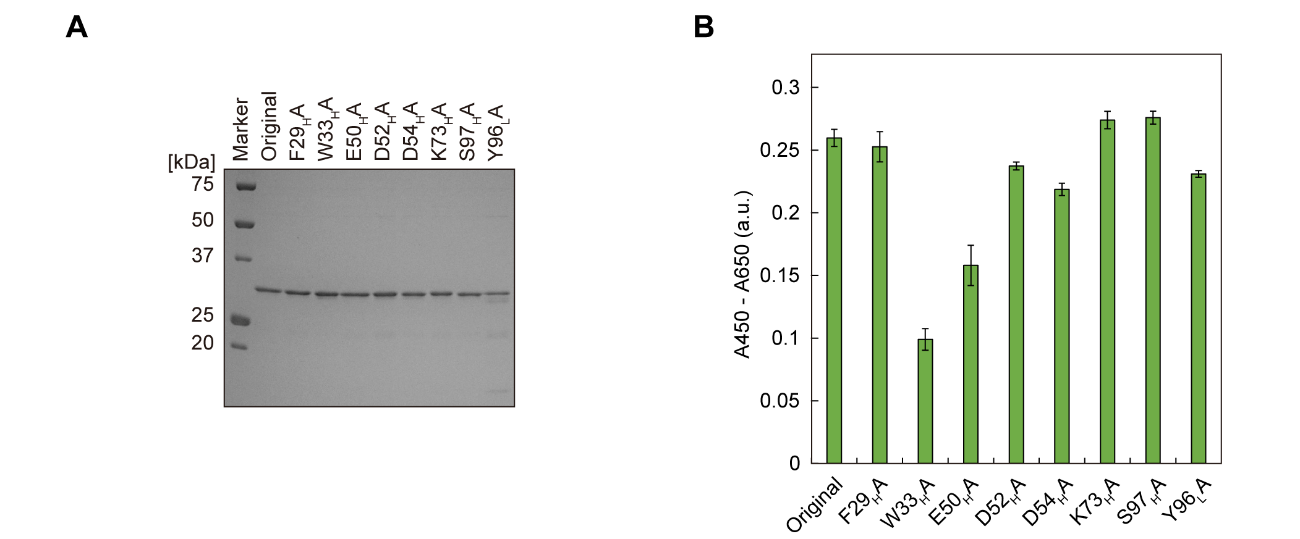


Figure S4. Preparation of Switchbody mutants. (A) SDS-PAGE analysis of purified Switchbody mutants. (B) Binding activity of Switchbody mutants to immobilized antigen, BGP-C11 examined by ELISA. Data are shown as mean ± standard deviation of triplicates. a.u.: arbitrary units.


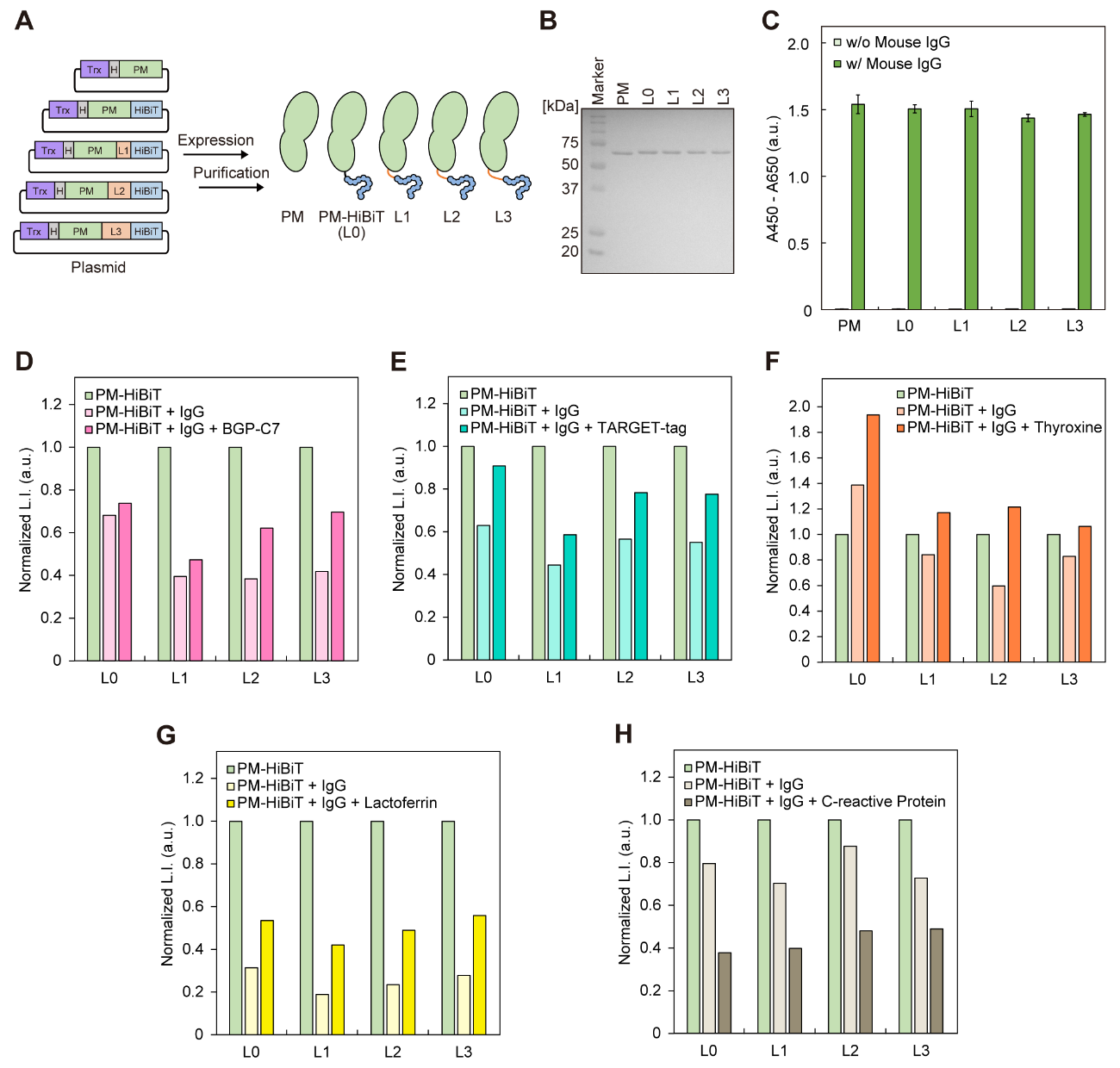


Figure S5. Preparation and characterization of PM-HiBiTs. (A) Schematic illustration of the preparation process for PM and PM-HiBiTs. L1: (G_3_S) linker, L2: (G_3_S)_2_ linker, L3: (G_3_S)_3_ linker, H: 6×His-tag, Trx: thioredoxin. (B) SDS-PAGE analysis of purified PM and PM-HiBiTs. (C) Binding activity of PM and PM-HiBiTs to immobilized mouse IgG examined by ELISA. Data are shown as mean ± standard deviation of triplicates. (D–H) Luminescence intensity of complexes of 1 nM PM-HiBiT and 5 nM IgG. The data are presented as a single measurement value for screening purposes only. L.I.: luminescence intensity. a.u.: arbitrary units.


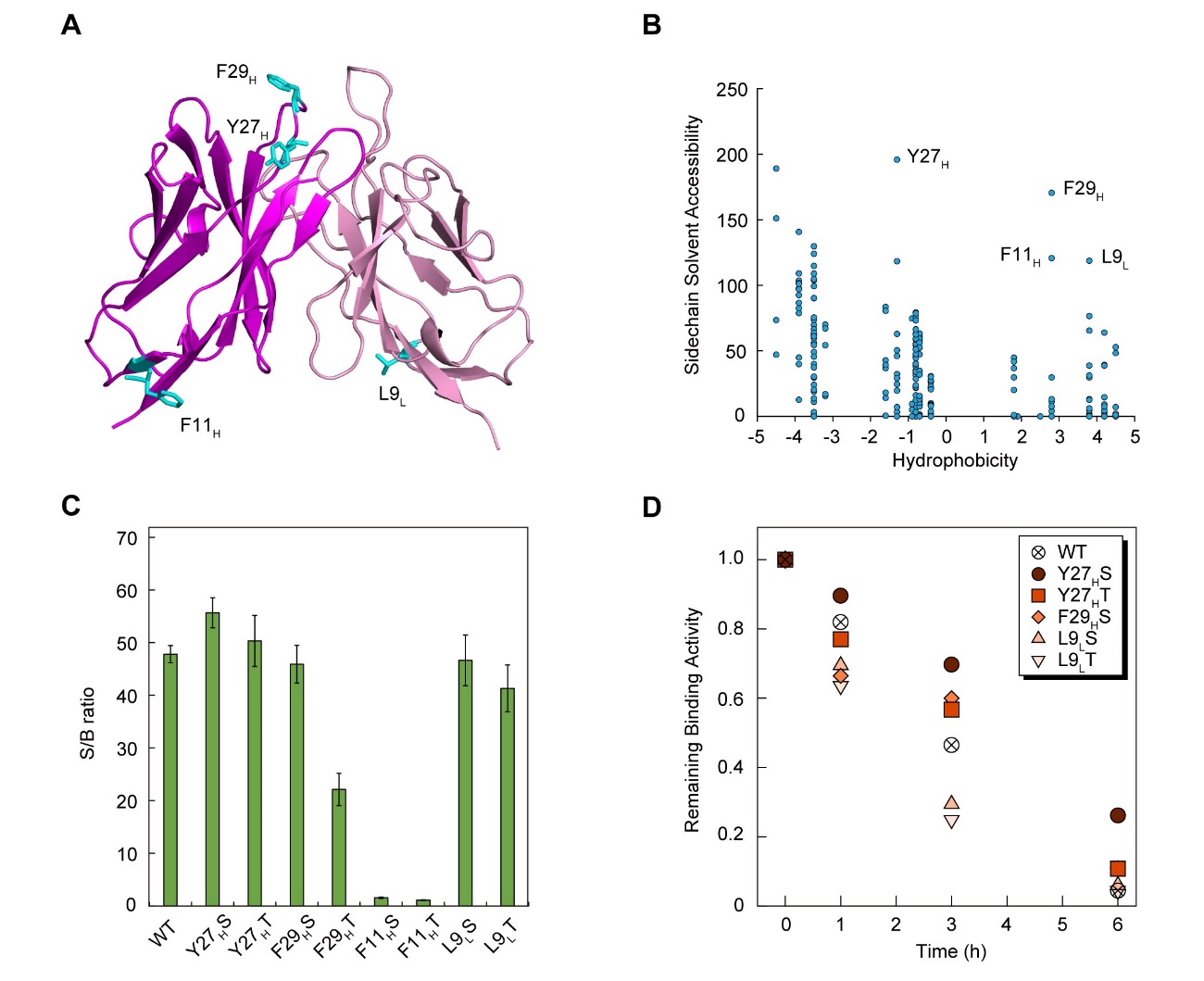


Figure S6. Preparation of thermostable scFv for NMR study. (A) Model structure of the scFv generated from the crystal structure (PDB ID: 5X5X) (VH in magenta, VL in pink, candidates for mutation in cyan). (B) Selection of hydrophobic amino acids with large solvent accessible surface area on the scFv. Selected amino acid was mutated to serine or threonine. (C) Binding activity of mutants to immobilized antigen, BGP-C11 examined by ELISA. S/B ratio were calculated from absorbance signal with antigen divided by without antigen. Data are shown as mean ± standard deviation of triplicates. (D) Remaining binding activity of mutants to immobilized antigen after 60°C with various incubation time. Data are shown as mean of triplicates.

**Movie S1.** MD simulation of Switchbody (L1) without antigen. The last 2.0 µs of trajectories from each of the five independent runs were sequentially connected. HiBiT is shown in blue, V_H_ in magenta, and V_L_ in pink.

**Movie S2.** MD simulation of Switchbody (L1) with antigen. The last 2.0 µs of trajectories from each of the five independent runs were sequentially connected. HiBiT is shown in blue, V_H_ in magenta, V_L_ in pink, and antigen peptide BGP-C7 in orange.

**References**

[46] C. Qian, A. Ninomiya, N. Shibukawa, H. Ueda, T. Yasuda, B. Zhu, T. Kitaguchi, “Detection of a large antigen through the masking and exposure of a fragment of split luciferase” *Anal. Sci.* **2025**, *41*, 857.

[47] W. Kabsch, “XDS” *Acta Cryst. D Biol. Crystallogr.* **2010**, *66*, 125.

[48] P. R. Evans, G. N. Murshudov, “How good are my data and what is the resolution?” *Acta Cryst. D Biol. Crystallogr.* **2013**, *69*, 1204.

[49] P. Emsley, B. Lohkamp, W. G. Scott, K. Cowtan, “Features and development of Coot” *Acta Cryst. D Biol. Crystallogr.* **2010**, *66*, 486.

[50] G. N. Murshudov, P. Skubák, A. A. Lebedev, N. S. Pannu, R. A. Steiner, R. A. Nicholls, M. D. Winn, F. Long, A. A. Vagin, “REFMAC5 for the refinement of macromolecular crystal structures” *Acta Cryst. D Struct. Biol.* **2011**, *67*, 355.

[51] J. Agirre, M. Atanasova, H. Bagdonas, C. B. Ballard, A. Baslé, J. Beilsten-Edmands, R. J. Borges, D. G. Brown, J. J. Burgos-Mármol, J. M. Berrisford, P. S. Bond, I. Caballero, L. Catapano, G. Chojnowski, A. G. Cook, K. D. Cowtan, T. I. Croll, J. É. Debreczeni, N. E. Devenish, E. J. Dodson, T. R. Drevon, P. Emsley, G. Evans, P. R. Evans, M. Fando, J. Foadi, L. Fuentes-Montero, E. F. Garman, M. Gerstel, R. J. Gildea, K. Hatti, M. L. Hekkelman, P. Heuser, S. W. Hoh, M. A. Hough, H. T. Jenkins, E. Jiménez, R. P. Joosten, R. M. Keegan, N. Keep, E. B. Krissinel, P. Kolenko, O. Kovalevskiy, V. S. Lamzin, D. M. Lawson, A. A. Lebedev, A. G. W. Leslie, B. Lohkamp, F. Long, M. Maly, A. J. McCoy, S. J. McNicholas, A. Medina, C. Millán, J. W. Murray, G. N. Murshudov, R. A. Nicholls, M. E. M. Noble, R. Oeffner, N. S. Pannu, J. M. Parkhurst, N. Pearce, J. Pereira, A. Perrakis, H. R. Powell, R. J. Read, D. J. Rigden, W. Rochira, M. Sammito, F. S. Rodríguez, G. M. Sheldrick, K. L. Shelley, F. Simkovic, A. J. Simpkin, P. Skubak, E. Sobolev, R. A. Steiner, K. Stevenson, I. Tews, J. M. H. Thomas, A. Thorn, J. T. Valls, V. Uski, I. Usón, A. Vagin, S. Velankar, M. Vollmar, H. Walden, D. Waterman, K. S. Wilson, M. D. Winn, G. Winter, M. Wojdyr, K. Yamashita, “The CCP4 suite: integrative software for macromolecular crystallography” *Acta Cryst. D Struct. Biol.* **2023**, *79*, 449.

[52] R. A. Laskowski, M. W. Macarthur, D. S. Moss, J. M. Thornton, “Procheck - a Program to Check the Stereochemical Quality of Protein Structures” *J. Appl. Crystallogr.* **1993**, *26*, 283.

[53] S. C. Lovell, I. W. Davis, W. B. Adrendall, P. I. W. de Bakker, J. M. Word, M. G. Prisant, J. S. Richardson, D. C. Richardson, “Structure validation by Cα geometry: φ,ψ and Cβ deviation” *Proteins Struct. Funct. Genet.* **2003**, *50*, 437.

[54] C. J. Williams, J. J. Headd, N. W. Moriarty, M. G. Prisant, L. L. Videau, L. N. Deis, V. Verma, D. A. Keedy, B. J. Hintze, V. B. Chen, S. Jain, S. M. Lewis, W. B. Arendall, J. Snoeyink, P. D. Adams, S. C. Lovell, J. S. Richardson, D. C. Richardson, “MolProbity: More and better reference data for improved all-atom structure validation” *Protein Sci.* **2018**, *27*, 293.

[55] A. Sali, T. L. Blundell, “Comparative Protein Modeling by Satisfaction of Spatial Restraints” *J. Mol. Biol.* **1993**, *234*, 779.

[56] W. L. Jorgensen, J. Chandrasekhar, J. D. Madura, R. W. Impey, M. L. Klein, “Comparison of Simple Potential Functions for Simulating Liquid Water” *J. Chem. Phys.* **1983**, *79*, 926.

[57] J. A. Maier, C. Martinez, K. Kasavajhala, L. Wickstrom, K. E. Hauser, C. Simmerling, “ff14SB: Improving the Accuracy of Protein Side Chain and Backbone Parameters from ff99SB” *J. Chem. Theory Comput.* **2015**, *11*, 3696.

[58] I. S. Joung, T. E. Cheatham, “Determination of alkali and halide monovalent ion parameters for use in explicitly solvated biomolecular simulations” *J. Phys. Chem. B* **2008**, *112*, 9020.

[59] R. Salomon-Ferrer, A. W. Götz, D. Poole, S. Le Grand, R. C. Walker, “Routine Microsecond Molecular Dynamics Simulations with AMBER on GPUs. 2. Explicit Solvent Particle Mesh Ewald” *J. Chem. Theory Comput.* **2013**, *9*, 3878.

[60] T. Darden, D. York, L. Pedersen, “Particle Mesh Ewald - an N.Log(N) Method for Ewald Sums in Large Systems” *J. Chem. Phys.* **1993**, *98*, 10089.

[61] H. J. C. Berendsen, J. P. M. Postma, W. F. Vangunsteren, A. Dinola, J. R. Haak, “Molecular-Dynamics with Coupling to an External Bath” *J. Chem. Phys.* **1984**, *81*, 3684.

[62] J.-P. Ryckaert, G. Ciccotti, H. J. C. Berendsen, “Numerical integration of the cartesian equations of motion of a system with constraints: molecular dynamics of *n*-alkanes” *J. Comput. Phys.* **1977**, *23*, 327.

[63] H. C. Andersen, “Rattle - a Velocity Version of the Shake Algorithm for Molecular-Dynamics Calculations” *J. Comput. Phys.* **1983**, *52*, 24.

[64] W. Humphrey, A. Dalke, K. Schulten, “VMD: Visual molecular dynamics” *J. Mol. Graph. Model.* **1996**, *14*, 33.

[65] R. T. McGibbon, K. A. Beauchamp, M. P. Harrigan, C. Klein, J. M. Swails, C. X. Hernández, C. R. Schwantes, L. P. Wang, T. J. Lane, V. S. Pande, “MDTraj: A Modern Open Library for the Analysis of Molecular Dynamics Trajectories” *Biophys. J.* **2015**, *109*, 1528.
